# Supplementary material for: Modular fluorescence complementation sensors for live cell detection of epigenetic signals at endogenous genomic sites
Source: Nat Commun. 2017 Sep 21;8:649. doi: 10.1038/s41467-017-00457-z (PMC5608954; doi:10.1038/s41467-017-00457-z)
Supplement: Supplementary file 2 — Supplementary Information [file 41467_2017_457_MOESM2_ESM.pdf]

Description of Supplementary Files

Title: Supplementary Information

Description: Supplementary Figures, Supplementary Tables, and Supplementary References

Title: Peer Review File

Supplemental figures and figure legends

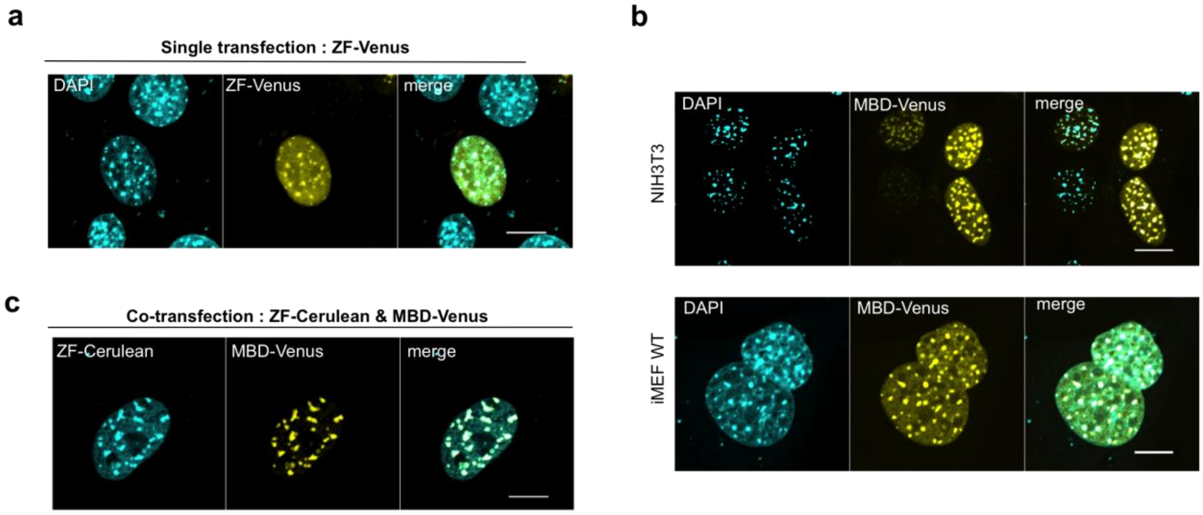

Supplementary Figure 1: Selection and validation of BiAD 1 sensor modules (**a**) Representative fluorescent microscopy image documenting the colocalization of the Venus-fused ZF with mouse pericentrometric heterochromatin, detected by DAPI co-staining. (**b**) Represenative fluorescent microscopy image documenting that the MBD-Venus fusion shows specific enrichment at DAPI-dense heterochromatic foci in NIH3T3 (top panel) and iMEF (bottom panel) cells. (**c**) Representative fluorescence microscopy image documenting that both the ZF and the MBD module can simultaneously bind at the same target sites as indicated by the co-localization of the BiAD modules in co-transfection experiments. Scale bar for all images is 10 µm. This figure refers to Fig. 2.

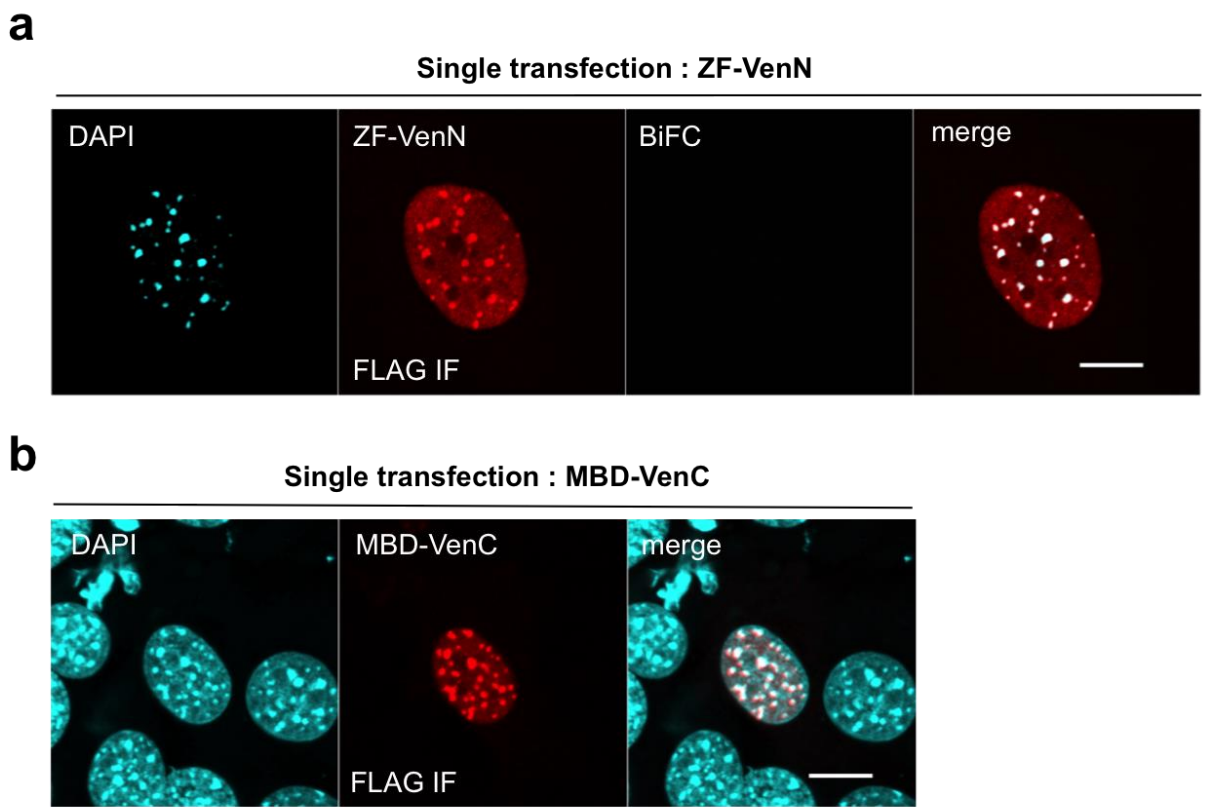

Supplementary Figure 2: Fusion of the BiAD 1 sensor modules with the split Venus fragments does not alter the cellular localization of the modules and does not give rise to background fluorescence. (**a**) Representative fluorescence microscopy image documenting that the split fluorophore-fused ZF module maintains its localization at DAPI dense foci. Furthermore, no residual fluorescence is observed in the BiFC channel documenting that the reconstitution of the fluorophore is mandatory for signal detection under these imaging settings. This is an important control since the Venus chromophore is located in the VenN part. The expression of the construct was detected by anti-FLAG immunofluorescence. **(b)** Anti-FLAG immunofluorescence image demonstrating that the 5mC specificity of the MBD is maintained upon its fusion with the VenC fragment as the construct maintains its localization to 5mC rich chromocenters. Scale bar is 10 µm. This figure refers to Fig. 2.

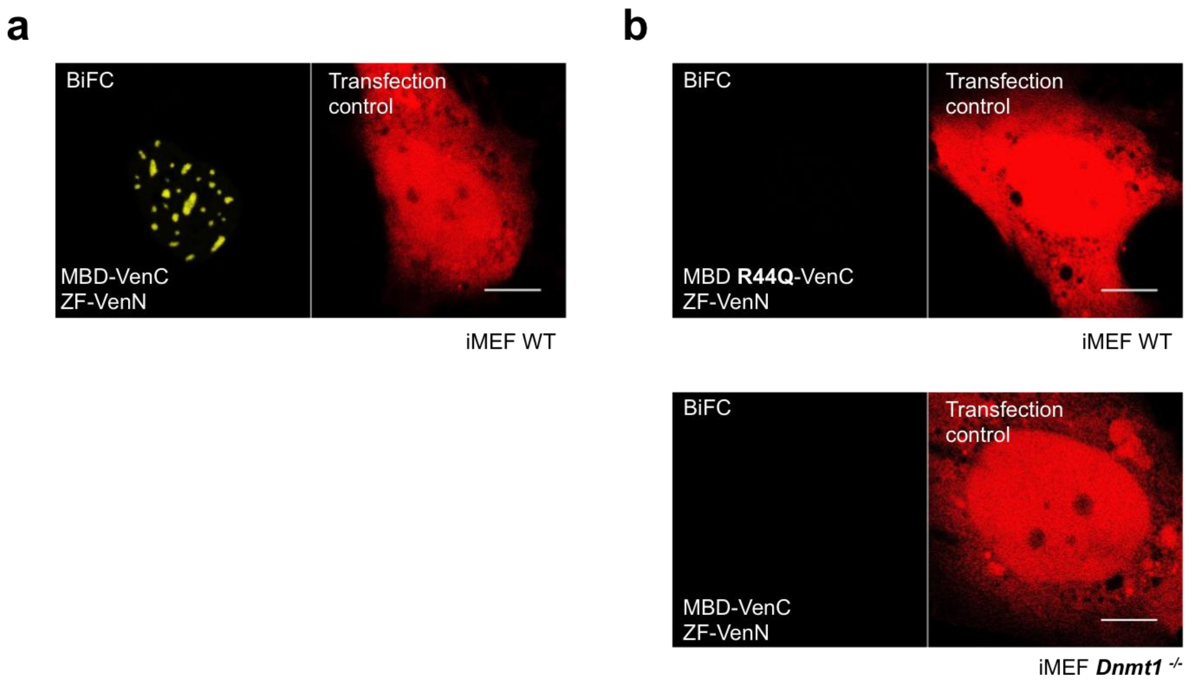

Supplementary Figure 3: The fluorescent signal produced by the BiAD sensor 1 is specific and can be detected by live cell microscopy. (**a**) Representative fluorescence microscopy image of live iMEF cells after transfection with the modules of the BiAD sensor 1. The bimolecular fluorescence complementation (BiFC) signal is shown in yellow. (**b**) Representative fluorescence microscopy images documenting the 5mC specificity of the BiAD 1 sensor under live cell imaging conditions. Upper panel: iMEF cells were transfected with the BiAD sensor where the MBD R44Q construct was used as detector module. Lower panel: DNMT1 deficient iMEF cells were transfected with the BiAD 1 modules. In both cases no signal is observed in the BiFC channel. Imaging was performed at 48 h after transfection. A plasmid encoding NLS-mRuby2 was used to identify transfected cells (red channel). Scale bar is 10 µm. This figure refers to Fig. 2.

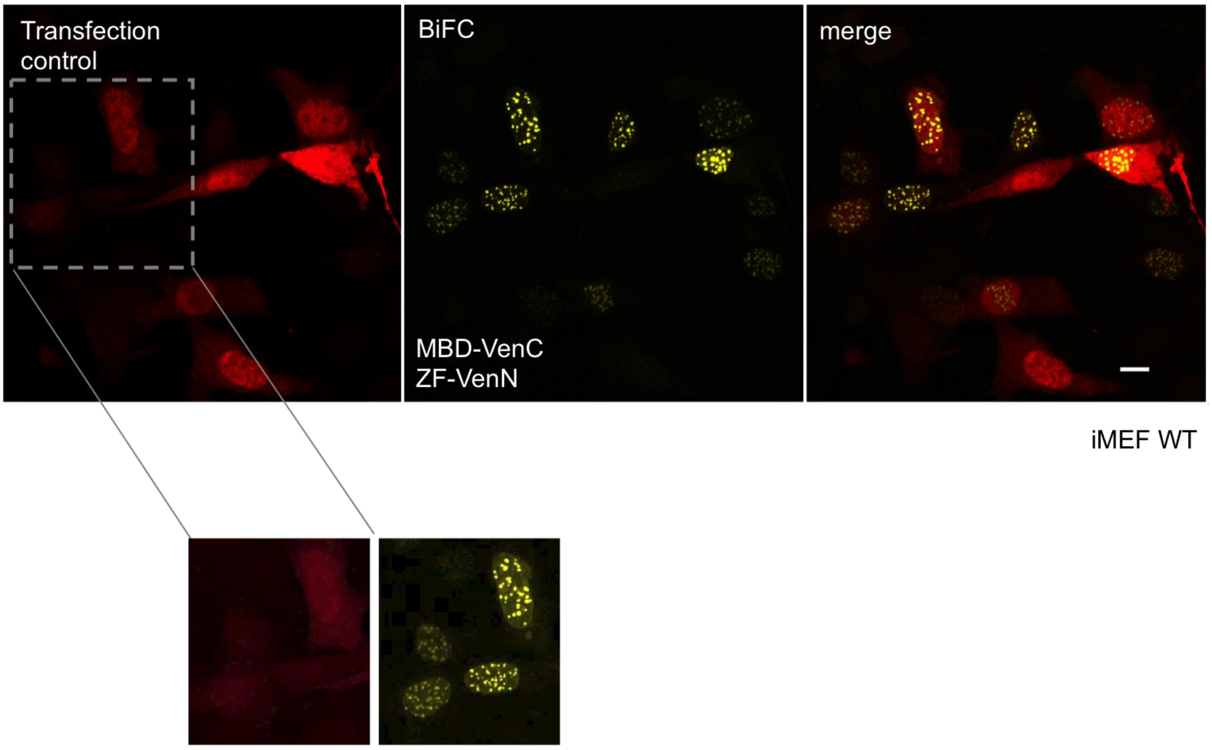

Supplementary Figure 4: BiAD sensor 1 shows a high fluorescence reconstitution yield when transfected in mouse fibroblasts. Zoom out fluorescence microscopy image documenting that BiAD sensor 1 gives rise to a strong BiFC signals in a large proportion of the transfected cells, identified based on the red fluorescence of the NLS-mRuby2 transfection marker. The modules of the sensor and the transfection marker were expressed from separate plasmids, leading to a lack of strong correlation between the intensity of the fluorescence observed in the transfection control channel and the BiFC yield (see also Methods section). The zoom in area shows a group of cells for which the contrast was adjusted such that the low NLS-mRuby2 expressing cells become visible. The transfection, imaging and display setting of this image are identical to those of Supplementary Figs. 6 and 8. This figure refers to Fig. 2. Scale bar 10 µm.

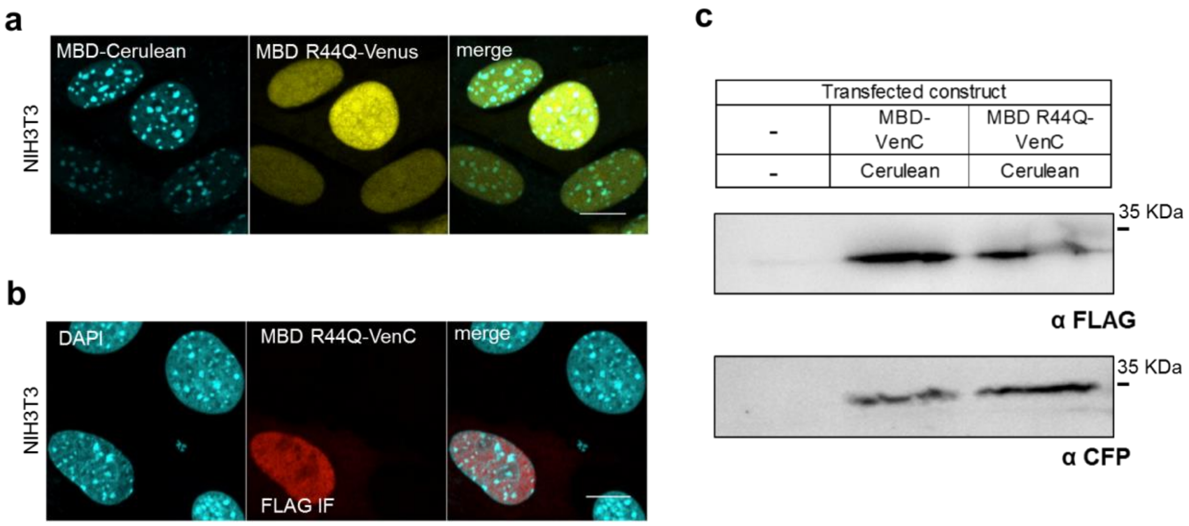

Supplementary Figure 5: Establishment of the MBD R44Q variant to validate the 5mC specificity of the DNA methylation biosensors. (**a**) Fluorescence microscopy images showing that the sub-nuclear localization pattern of the R44Q MBD domain mutant in mouse fibroblasts differs from that of the WT. (**b**) Anti-FLAG immunofluorescence images demonstrating that the diffuse localization of the MBD R44Q, thus its lack of binding pericentromeric 5mC marks, is maintained upon fusing the domain with the VenC fragment. (**c**) The R44Q mutation does not influence the expression and protein stability of the MBD-VenC fusion, as determined by Western blot with lysates of transfected cells. Co-transfection with mCerulean was used to normalize for differences in transfection efficiency. For detection of mCerulean expression, the same volumes of lysates as used in the anti-FLAG western blot were loaded on a separate SDS-PAGE. Lysate of un-transfected cells was loaded in the first lane. Scale bar for all images is 10 µm. This figure refers to Fig. 2.

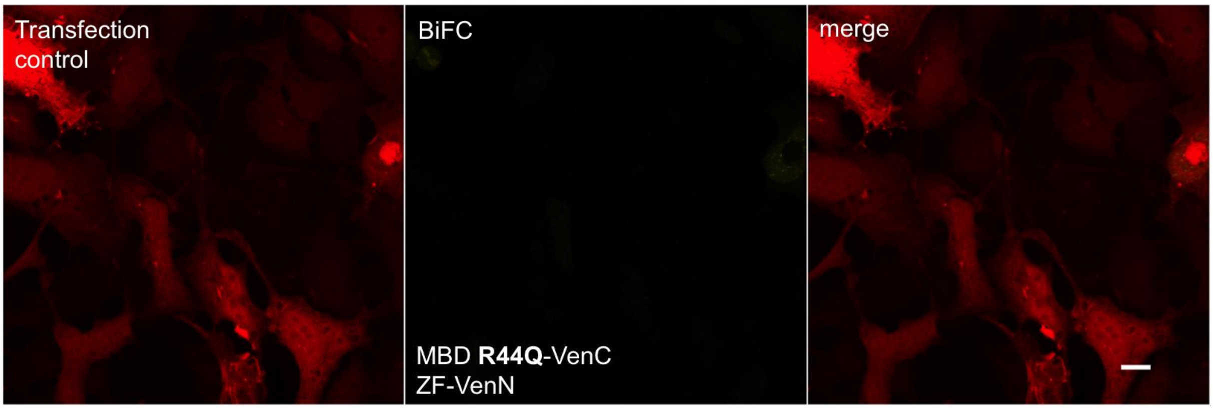

Supplementary Figure 6: Validation of the 5mC specificity of the BiAD sensor 1 by using the MBD R44Q variants as a detector module. Zoom out fluorescence microscopy image documenting that BiAD sensor 1 gives rise to only very weak BiFC signals when the R44Q variant is used instead of the WT MBD as a detector module. NLS-mRuby2 was used to identify transfected cells (red channel). The transfection, imaging and display setting of this image are identical to those of Supplementary Figs. 4 and 8. Scale bar 10 µm. This figure refers to Fig. 2.

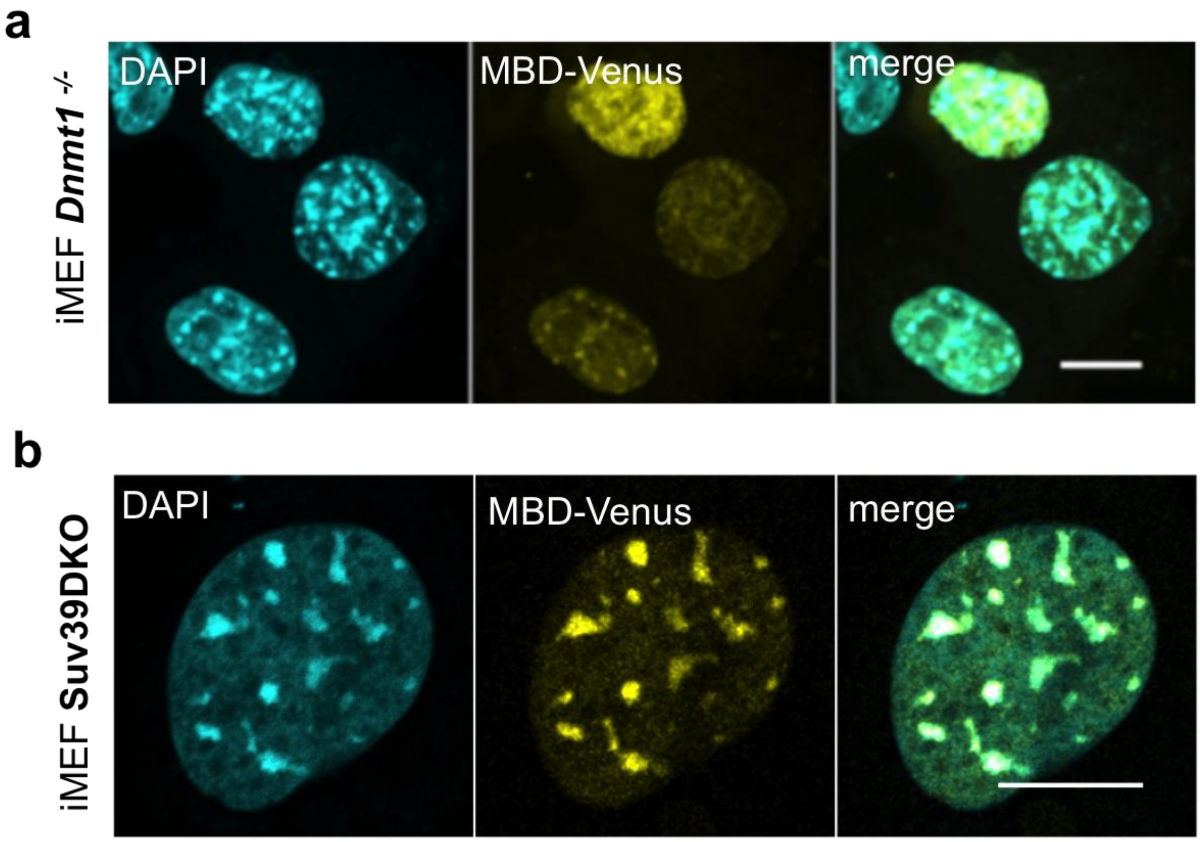

Supplementary Figure 7: Quality control of the 5mC detector module. (a) In DNMT1-deficient iMEF cells, the MBD of MBD1 is no longer enriched at DAPI stained pericentromeric repeats. (**b**) By contrast, in Suv39H1 and H2 double knock out cells (Suv39DKO) which are depleted in H3K9me3 but retain high 5mC levels [1,2], no changes in the localization of the MBD domain were observed. This indicates the localization of the MBD detector is directly dependent on the presence of the 5mC modification and is not influenced by chromatin organisation. Scale bar 10 µm. This figure refers to Fig. 2.

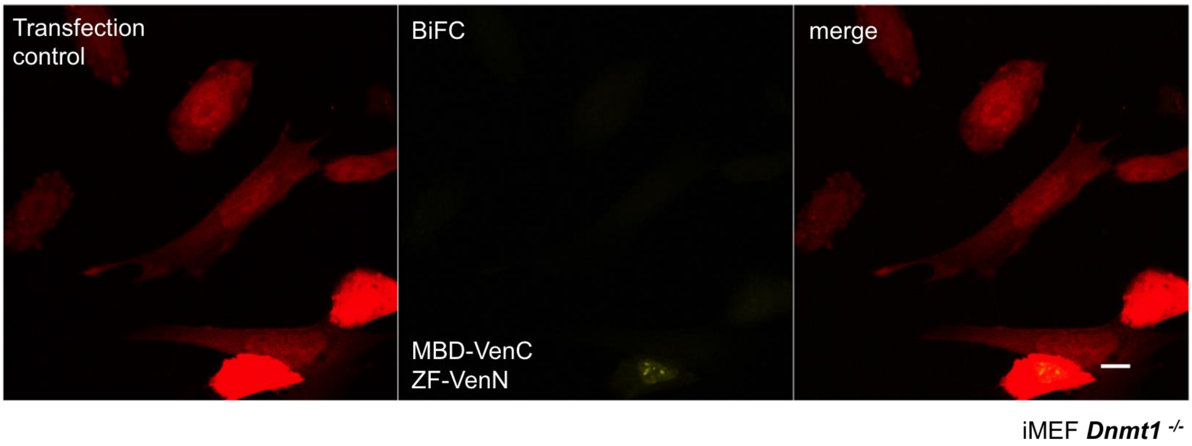

Supplementary Figure 8: Validation of the 5mC specificity of BiAD sensor 1 in DNMT1 deficient mouse fibroblasts. Zoom out fluorescence microscopy image documenting that BiAD sensor 1 only gives rise to very weak BiFC signals when transfected into cells with reduced DNA methylation. NLS-mRuby2 was used to identify transfected cells (red channel). The transfection, imaging and display setting of this image are identical to those of Supplementary Figs. 4 and 6. Scale bar 10 µm. This figure refers to Fig.2.

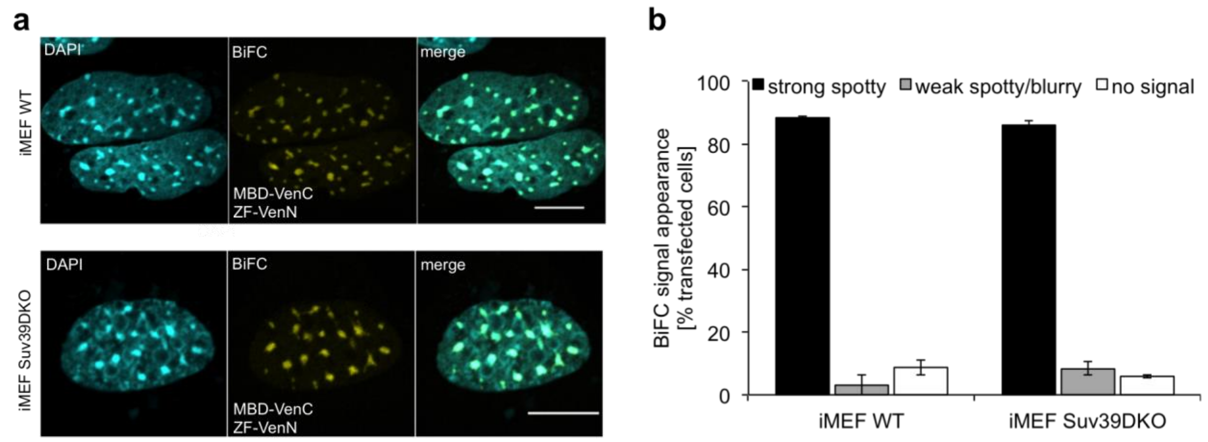

Supplementary Figure 9: 5mC readout by the BiAD sensor 1 is independent of changes in H3K9me3 levels. (**a**) Fluorescence microscopy images documenting comparable strong BiFC signals of the 5mC-specific BiAD sensor 1 in WT iMEF (top panel) and Suv39DKO iMEF cells (bottom panel). Scale bar for all images is 10 µm. The cells were fixed 48 h after transfection, the imaging and display setting are identical between the 2 panels of the figure. (**b**) Quantification of the experiments representatively shown in panel a. For details cf. Supplementary Tables 1, 6 and 7. Figure refers to Figs. 2d and 6c. Error bars show the s.e.m. of two independent biological replicated.

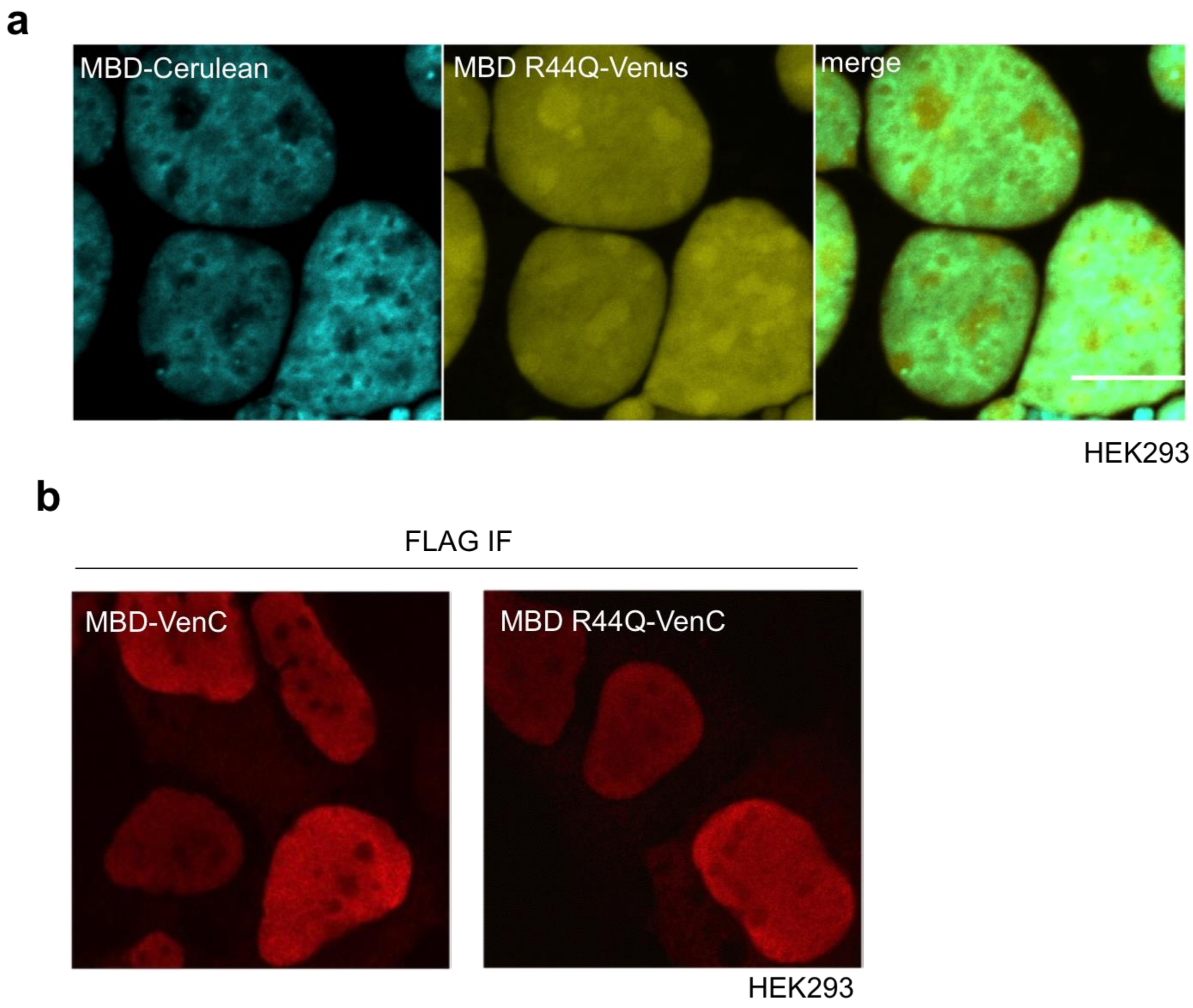

Supplementary Figure 10: The 5mC sensor used in BiAD sensor 1 can be incorporated in BiAD sensor 2. (**a**) Fluorescence microscopy image showing that the sub-nuclear localization pattern of the R44Q MBD domain mutant in HEK293 cells differs from that of the WT protein similarly as observed in NIH3T3 cells (Supplementary Fig.5a). (**b**) Anti-FLAG immunofluorescence images demonstrating that the VenC fusion does not affect the localization of the WT (left panel) or the R44Q (right panel) MBD domain in HEK293 cells. Scale bar 10 µm. This figure refers to Fig. 3.

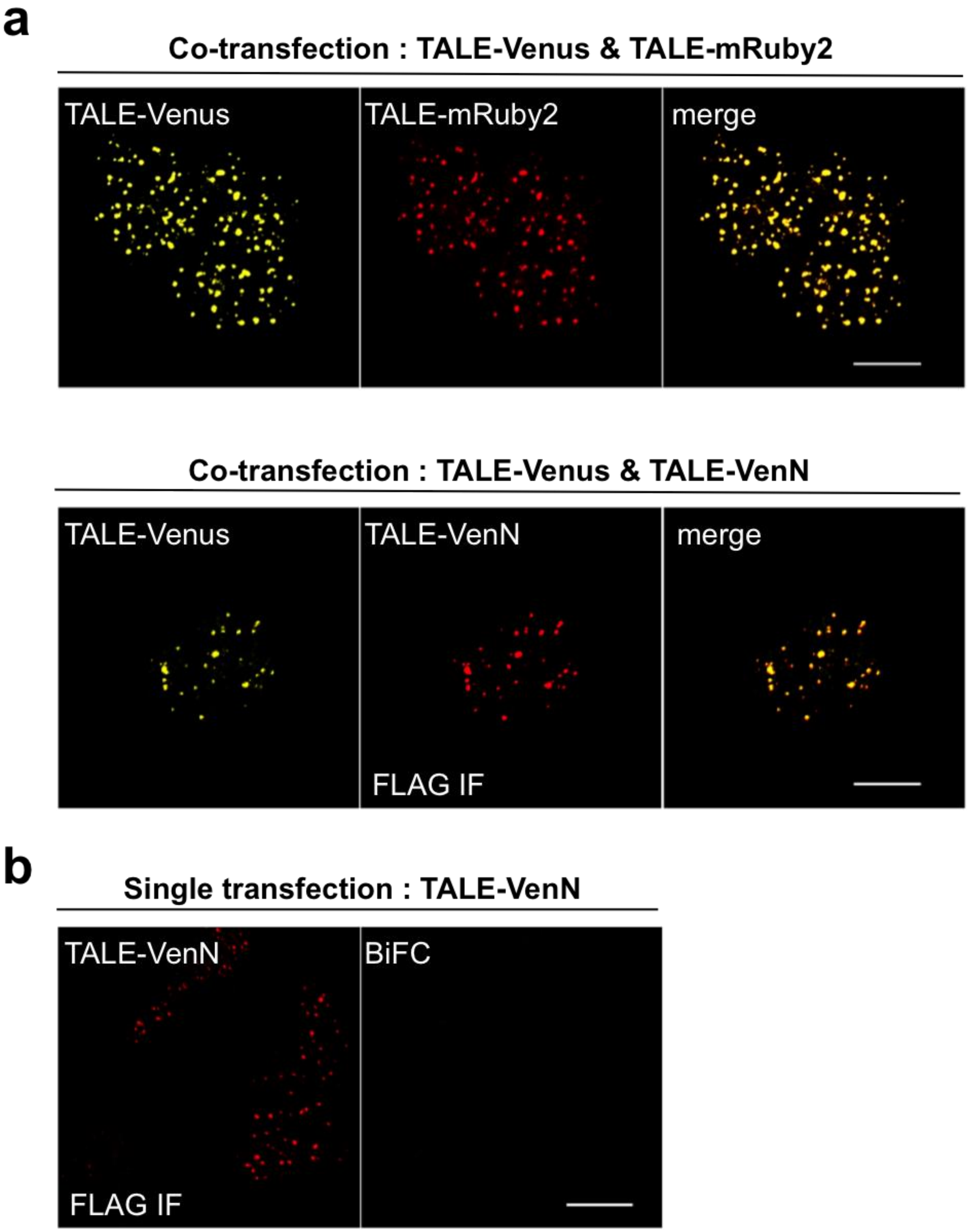

Supplementary Figure 11: Quality control of the TALE DNA-binding domain used in BiAD sensor 2. (**a**) Fluorescence microscopy image documenting the co-localization of the TALE-mRuby2 construct generated in this study with the TALE-Venus described and validated previously by Ma et al., 2013. Fusing of the DNA binding domain with mRuby2 instead of Venus does not lead to an alteration in the localization of the construct indicating that the construct validation performed by Ma et al., 2013 can be extrapolated to the mRuby2 fusion. (**b**) Representative anti-FLAG

immunofluorescence image showing that the TALE-VenN fusion maintains its DNA sequence specificity as indicated by the co-localization of this construct with TALE-Venus. (**c**) Fluorescence microscopy image documenting the absence of a BiFC signal in cells transfected only with TALE-VenN. The expression of the construct was detected by anti-FLAG immunofluorescence. Scale bar 10 µm for all images. This figure refers to Fig. 3.

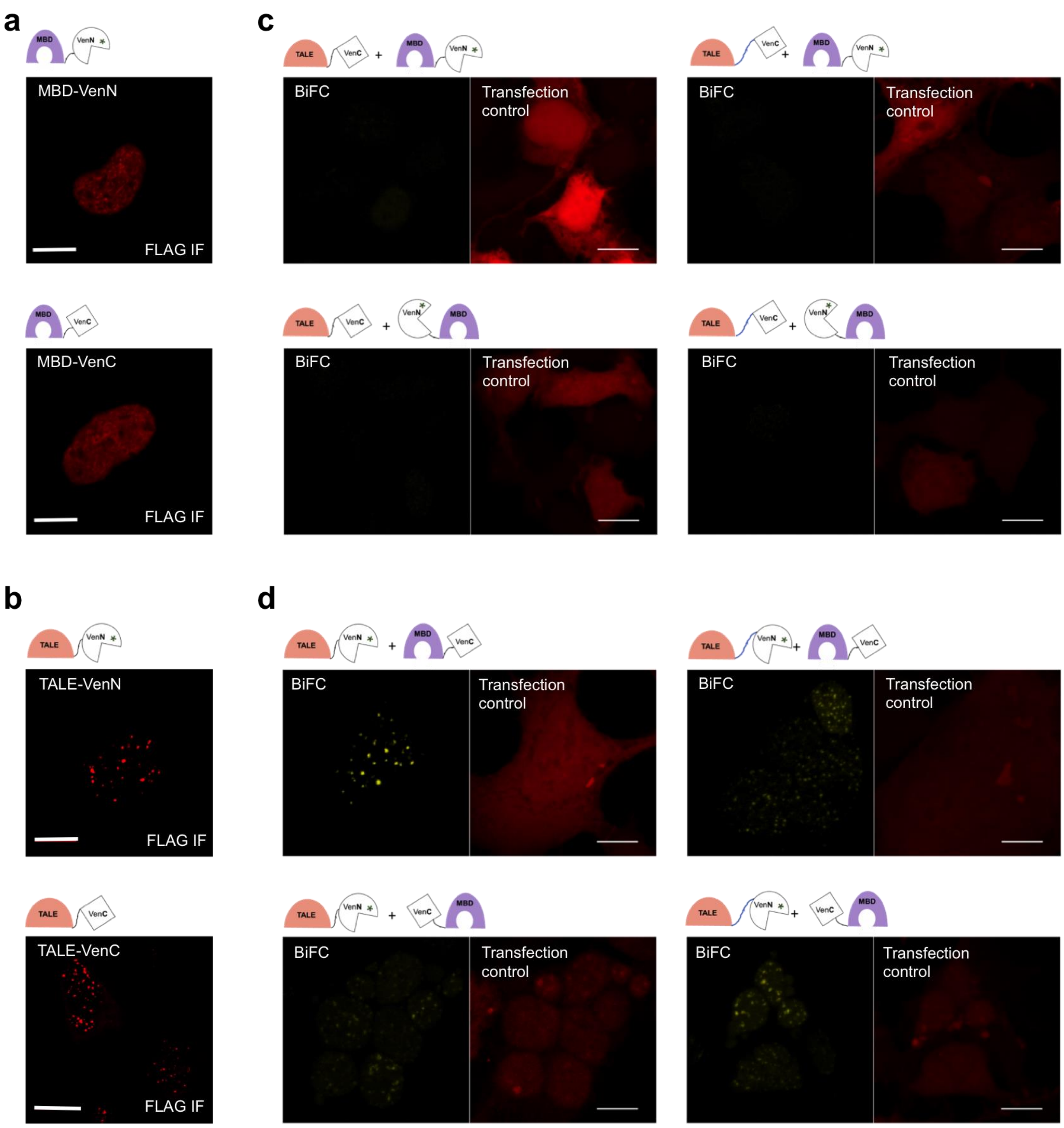

Supplementary Figure 12: Optimization of the BiFC output of the BiAD sensor 2 through fluorophore repositioning. (**a**) Immunofluorescence images documenting that fusion with VenN or VenC does not lead to the aggregation of the MBD construct (top vs. bottom panel). (**b**) VenN and VenC fusions of the TALE module (top vs. bottom panel) displayed a spotty localization in immunofluorescence images. This was comparable to what was observed for the TALE-Venus fusion. (**c**) Fluorescence microscopy images showing representative outcomes of the VenN fluorophore rearrangement around the MBD detector aiming to increase the sensitivity of the BiAD sensor 2. The VenC domain was fused to the TALE anchor, and separated either through a 7 amino acid (shown in black) or an 18 amino acid long linker (shown in blue). The lack of BiFC signal could not be attributed to miss-folding or delocalization of the VenN-MBD fusions, as immunofluorescence images showed a granular nuclear localization of all split-fluorophore fusions similar as with the full fluorophore-fused domain (Supplementary Figs. 12a and 10) (**d**) Fluorescence

microscopy images showing representative outcomes of the VenC fluorophore rearrangement on the MBD detector aiming to increase the sensitivity of the BiAD sensor 2. The VenN part was fused to the TALE anchor, and separated through either a 7 (shown in black) or 18 amino acid long linker (shown in blue). The tested combinations are schematically shown on top of the corresponding image. The transfection, imaging and display setting of this image are identical between cells shown in panels c-d and a-b, respectively. All cells were fixed at 48 h after transfection. Scale bar is 10 µm. The figure refers to Fig. 3a.

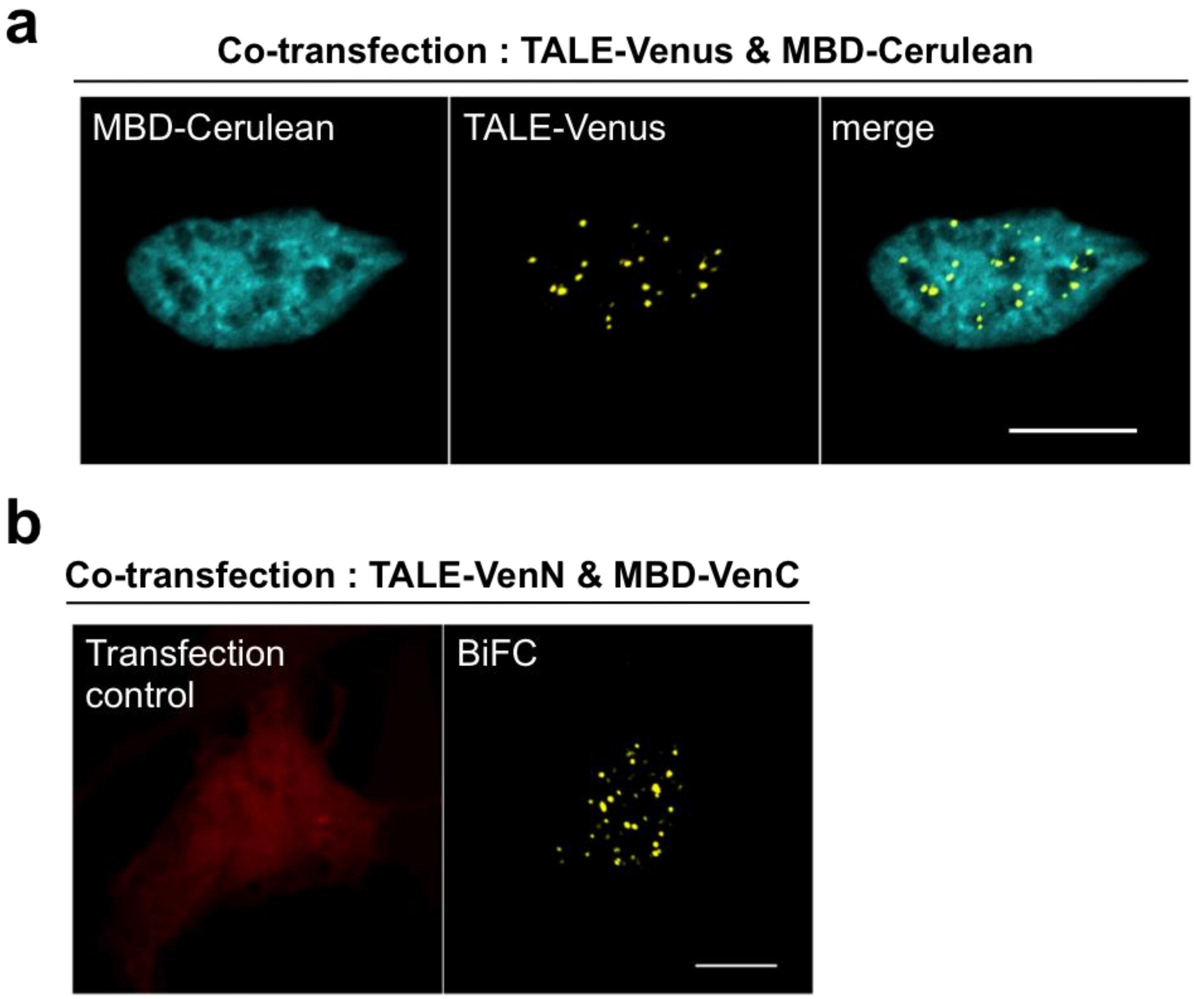

Supplementary Figure 13: Comparison between the BiAD approach and co-localization microscopy for 5mC detection at human centromeric sites. (**a**) Representative fluorescence microscopy image showing the localization of the full fluorophore tagged MBD and TALE modules in HEK293 cells. The merge image documents the overlap between the fluorescence of the TALE and MBD signals. (**b**) Representative fluorescence microscopy image documenting the localization of the BiFC signal in human cells. Comparison between the merged image in panel a and the pattern observed for the BiAD signal, highlights the advantage of the BiAD technology over co-localization microscopy. Scale bar for all images is 10 µm. This figure refers to Fig. 3.

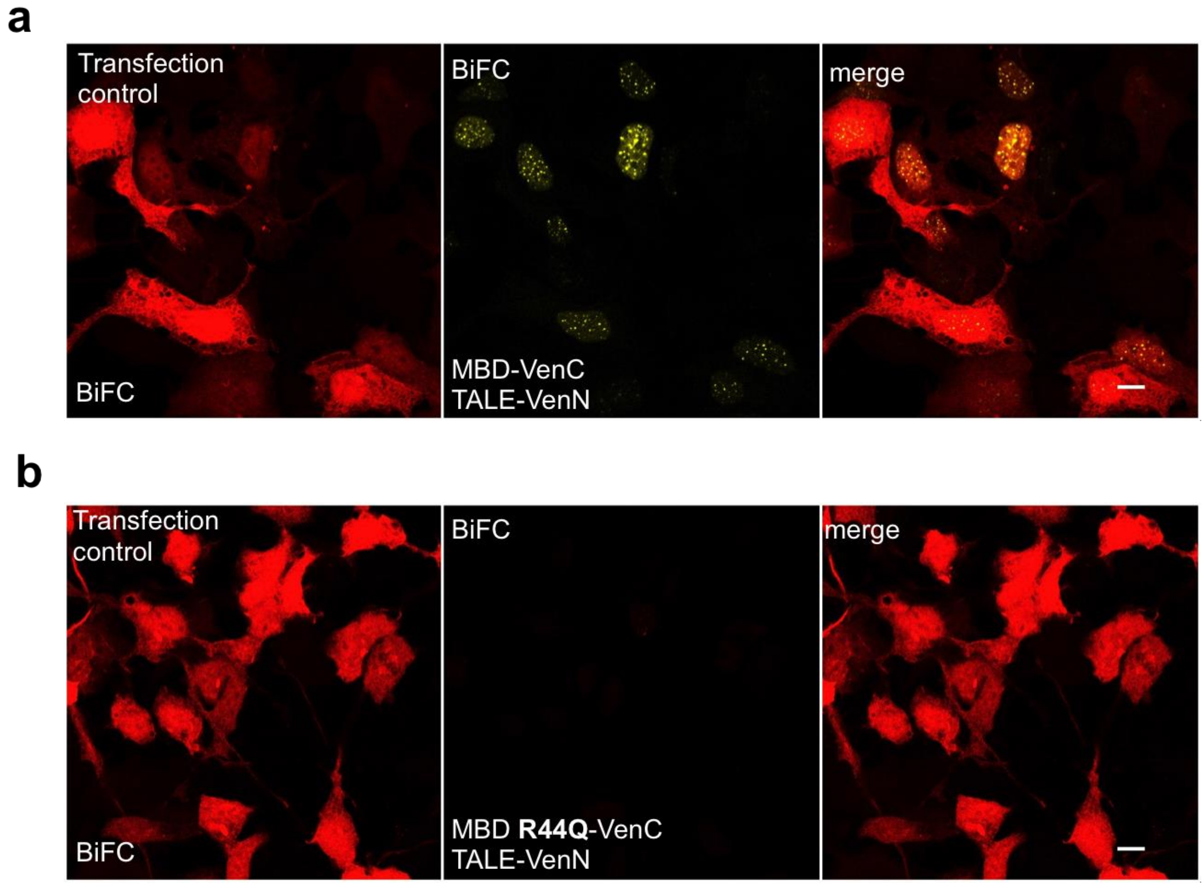

Supplementary Figure 14: Validation of the 5mC specificity of the BiAD sensor 2. (**a**) Zoom out fluorescence microscopy images documenting that the BiAD sensor 2 leads to a strong BiFC signal only when an intact MBD domain is used. **(b)** Replacing this with the R44Q variant, dramatically reduced the BiFC yield. The transfection, imaging and display setting of this image are identical between the cells shown in panels a and b. Images were taken 48 h after transfection. Scale bar is 10 µm. This figure refers to Fig. 2.

a

GTTATTGGATATTTGGAGTATTTTGAGGTTTATGGTGGAAAAGGAAATATTTTTATAAAAAAAAATT**AGACAG**
AAGCATTCTCAGAAATAT**CG**TTTTGATGTGTGAATTTATTTTATAGAGTTGAATTTTTTTTTTTTTTTTTTTTTT
TGAGATGGAGTTT**CG**TTTTGT**CG**TTTAGGT**CG**GATTG**CG**GATTGTAGTGGTTTAATTTTAGTTTATTGTAAGT
TT**CG**TTTTT**CG**GGTTTATGTTATTTTTTTGTTTTAGTTTTT**CG**AGTAGTTGGGATTATAGGTGTT**CG**TTAT**CGC**
GTTTAGTTAATTTTATGTATTTTTAGTAGAGA**CG**GGGTTTTATTTTGTTAGTTAGGATGGTTT

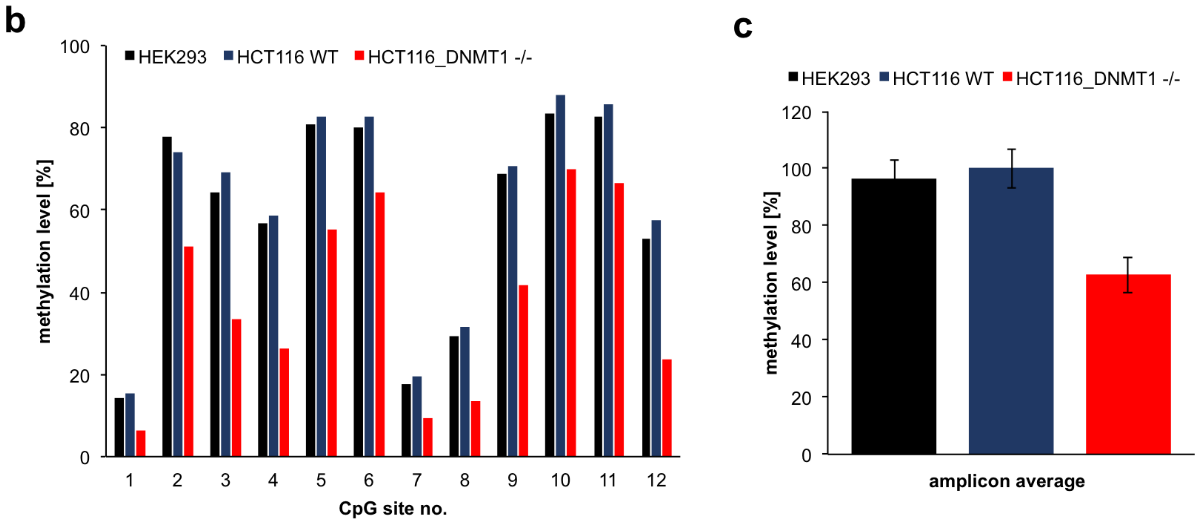

Supplementary Figure 15: TALE binding sites (TBS) show reduced DNA methylation in HCT116 cells that express a hypomorphic DNMT1 variant. (**a**) The sequence of the 358bp amplicon used for NGS. The TBS is highlighted in green, while each of the 12 CG sites is annotated in red. (**b**) Average DNA methylation at each of the 12 CG sites within the amplicon shown in panel a. All sites show a clear reduction in DNA methylation levels in the DNMT1 hypomorphic cell line. (c) Average DNA methylation over all CG sites shown in panel b. In line with the data shown in panel b, a strong reduction in DNA methylation was detectable at all 12 CG sites within the sequenced amplicon. Error bars represent s.e.m. of the methylation variation between the individual CG sites. This figure refers to Fig. 4.

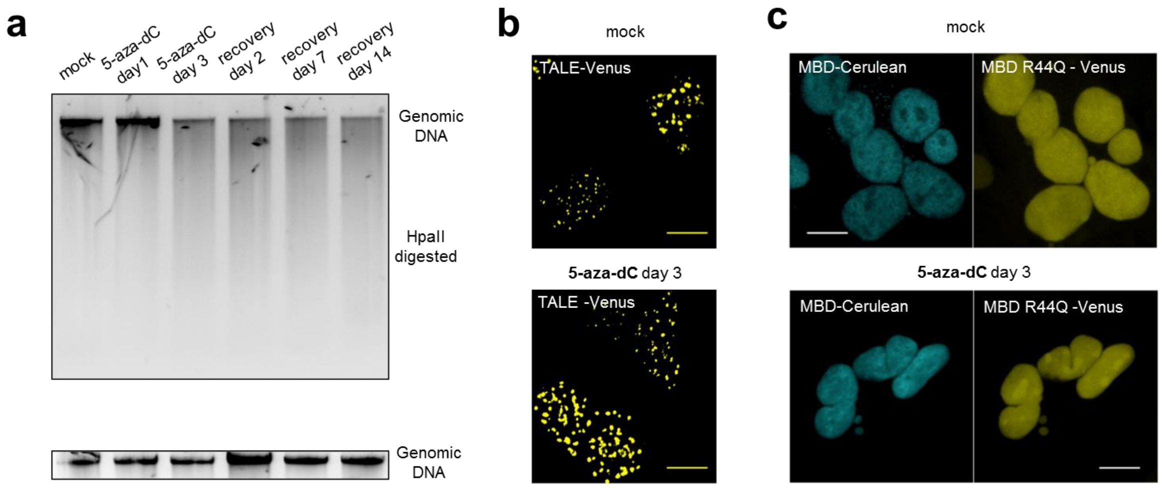

Supplementary Figure 16: Assessment of the efficiency of the 5-aza-dC treatment and the effect of the drug on the cellular localization of the domains used for BiAD sensor 2. (**a**) Agarose gel documenting the efficiency of the 5-aza-dC treatment. Genomic DNA was collected at the annotated time points and digested with the methylation sensitive restriction enzyme HpaII. Equal amounts of undigested DNA were used as loading control. Dramatic global demethylation was observed after 3 days of treatment and reduced methylation could still be detected 14 days after drug removal. (**b**) Representative fluorescence microscopy images documenting that the localization of the TALE anchor in HEK293 cells is not affected by the 5-aza-dC treatment. (**c**) Fluorescence microscopy images showing the localization of the MBD 5mC-detecting module in mock (top panel) and 5-aza-dC-treated (bottom panel) cells. Scale bar for all images is 10 µm. The figure refers to Fig. 4.

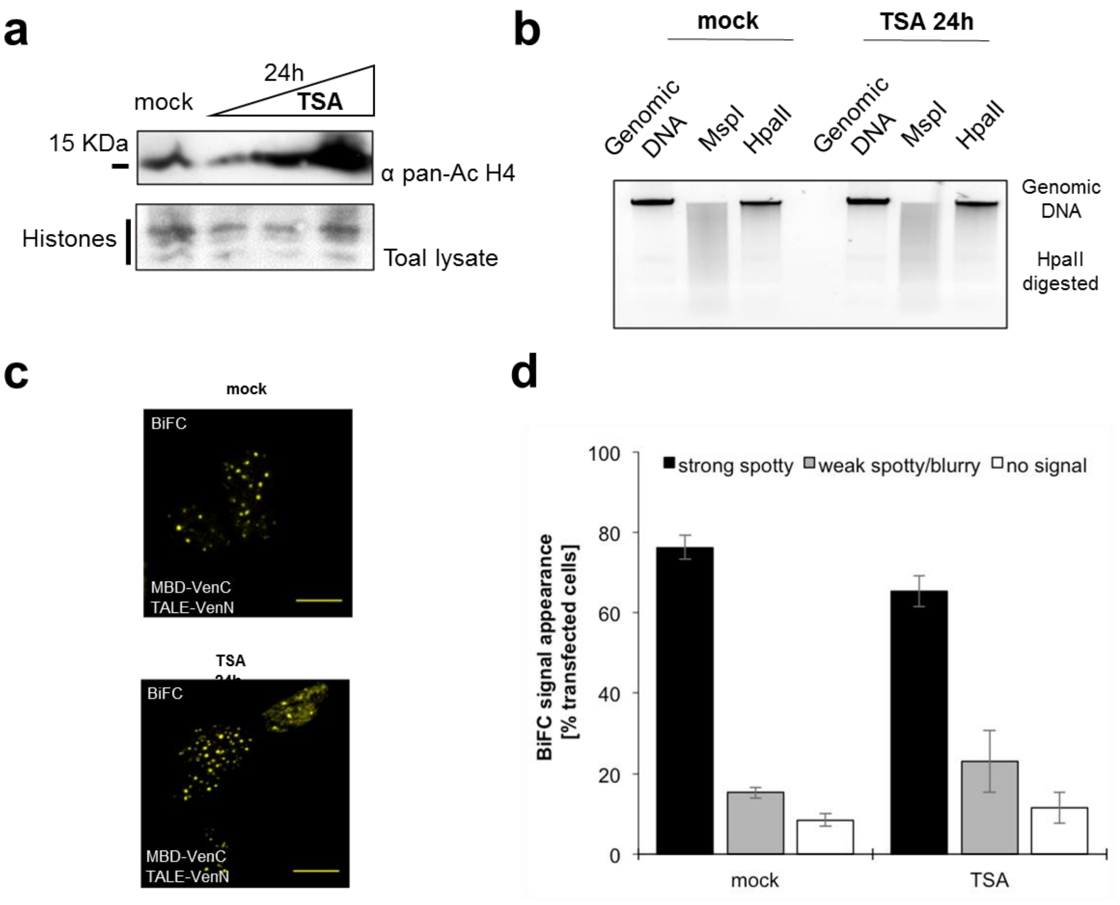

Supplementary Figure 17: Quality control of the TSA treatment and evaluation of its effect on the fluorescent signal produced by BiAD sensor 2. (**a**) Western blot validation of the increase in H4Ac in total cell lysates isolated from HEK293 cells after treatment with increasing amounts of TSA. TSA is a potent inhibitor of histone deacetylases and was shown to cause dramatic genome-wide chromatin decondensation [3]. The highest TSA concentration was selected for further experiments. (**b**) Agarose gel of genomic DNA isolated from mock and TSA-treated cells. HpaII DNA methylation-sensitive digest patterns do not show global differences in DNA methylation levels between the two samples. Corresponding amounts of undigested and MspI-digested DNA were used as loading controls. (**c**) Fluorescence microscopy images demonstrating that the BiAD sensor 2 is not affected by TSA-induced alteration of the local chromatin compaction state in cells. Scale bar for all images is 10 µm. The transfection, imaging and display setting of the BiFC images displayed are identical. **(d)** Quantification of the number of cells with BiFC signal in the experiments shown in panel c. Error bars in all images represent the s.e.m. for two biological replicates (for details cf. Supplementary Tables 2, 6 and 7). The figure refers to Fig. 4.

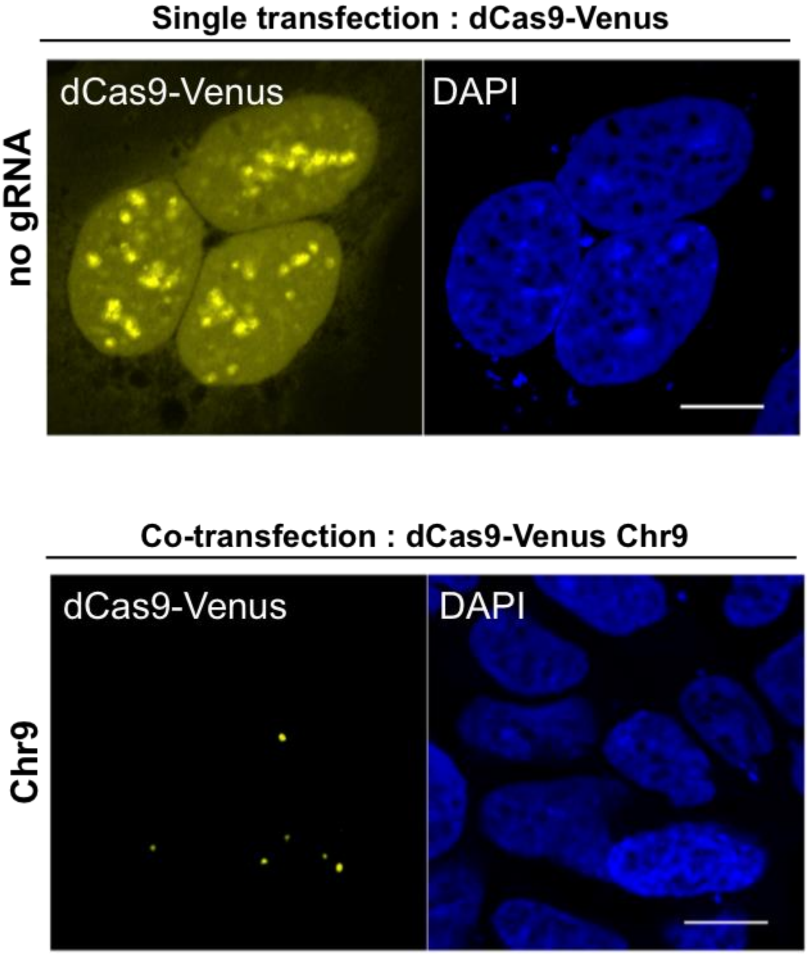

Supplementary Figure 18: Quality control of the DNA-binding anchor modules used in BiAD sensor 3. Fluorescence localization images documenting the DNA sequence specificity and guide RNA-dependence of the dCas9-Venus fusion. A clear spotty localization of dCas9-Venus was only observed after co-transfection with a guide RNA targeting a repetitive DNA sequence unique to chromosome 9 (top vs bottom panel). Scale bar for all images is 10 µm. This figure refers to Fig. 5.

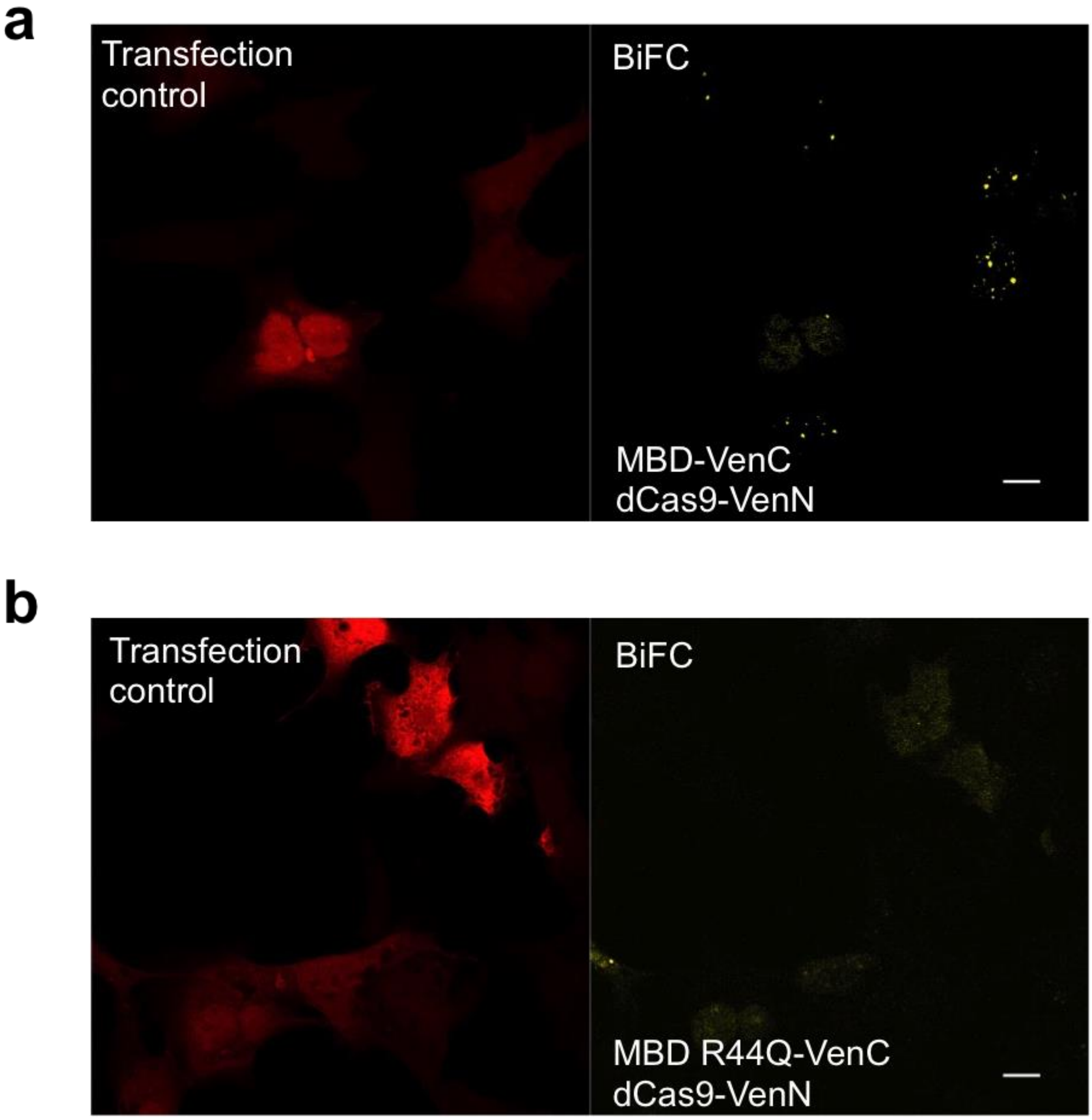

Supplementary Figure 19: Validation of the 5mC specificity of the BiAD sensors 3. (**a**) Zoom out fluorescence microscopy images documenting that the BiAD sensor 2 leads to a strong BiFC signal only when an intact MBD domain is used. **(b)** Replacing this with the R44Q variant, dramatically reduced the BiFC yield. The transfection, imaging and display setting of this image are identical between the cells shown in panel a and b. Scale bar for all images is 10 µm. This figure refers to Fig. 5.

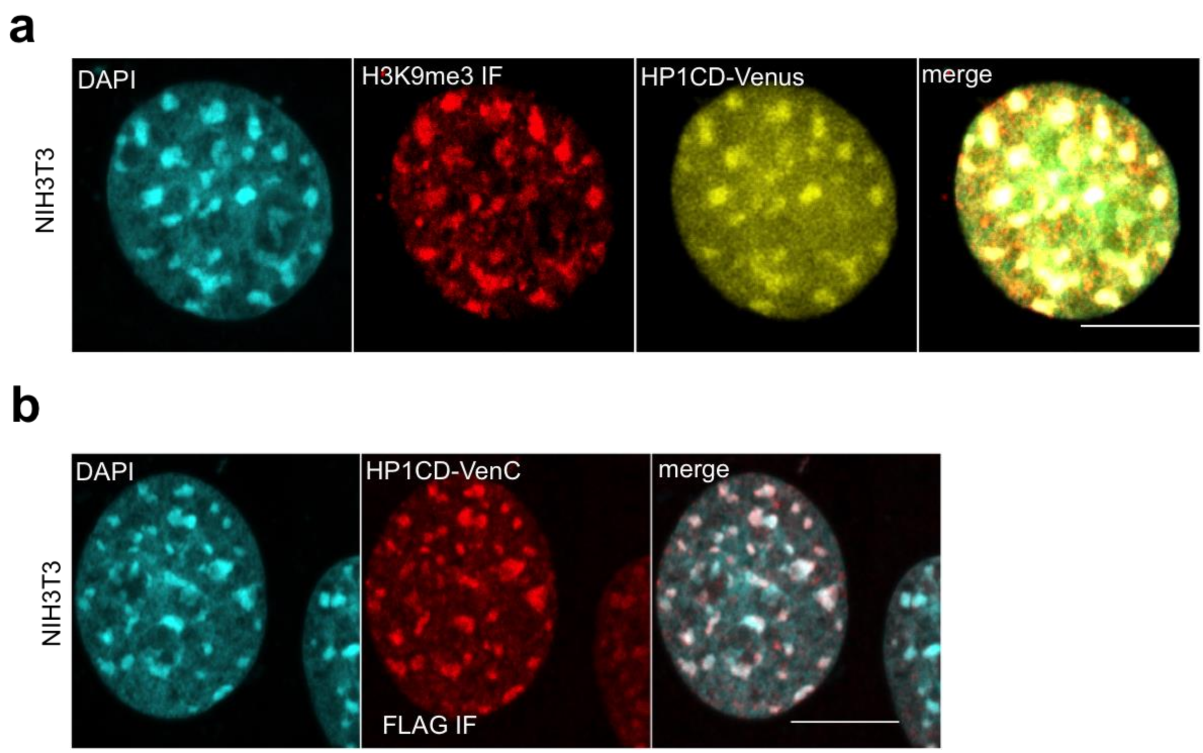

Supplementary Figure 20: Quality control of the H3K9me3 detector module used in this study. (**a**) Fluorescence microscopy image documenting the H3K9me3 specificity of the HP1CD-Venus domain, as indicated by the overlap of the domain with the antibody staining signal. (**b**) The HP1CD-VenC detector domain specifically localizes to mouse DAPI chromocenters in mouse fibroblasts. Scale bar for all images is 10 µm. This figure refers to Fig. 6.

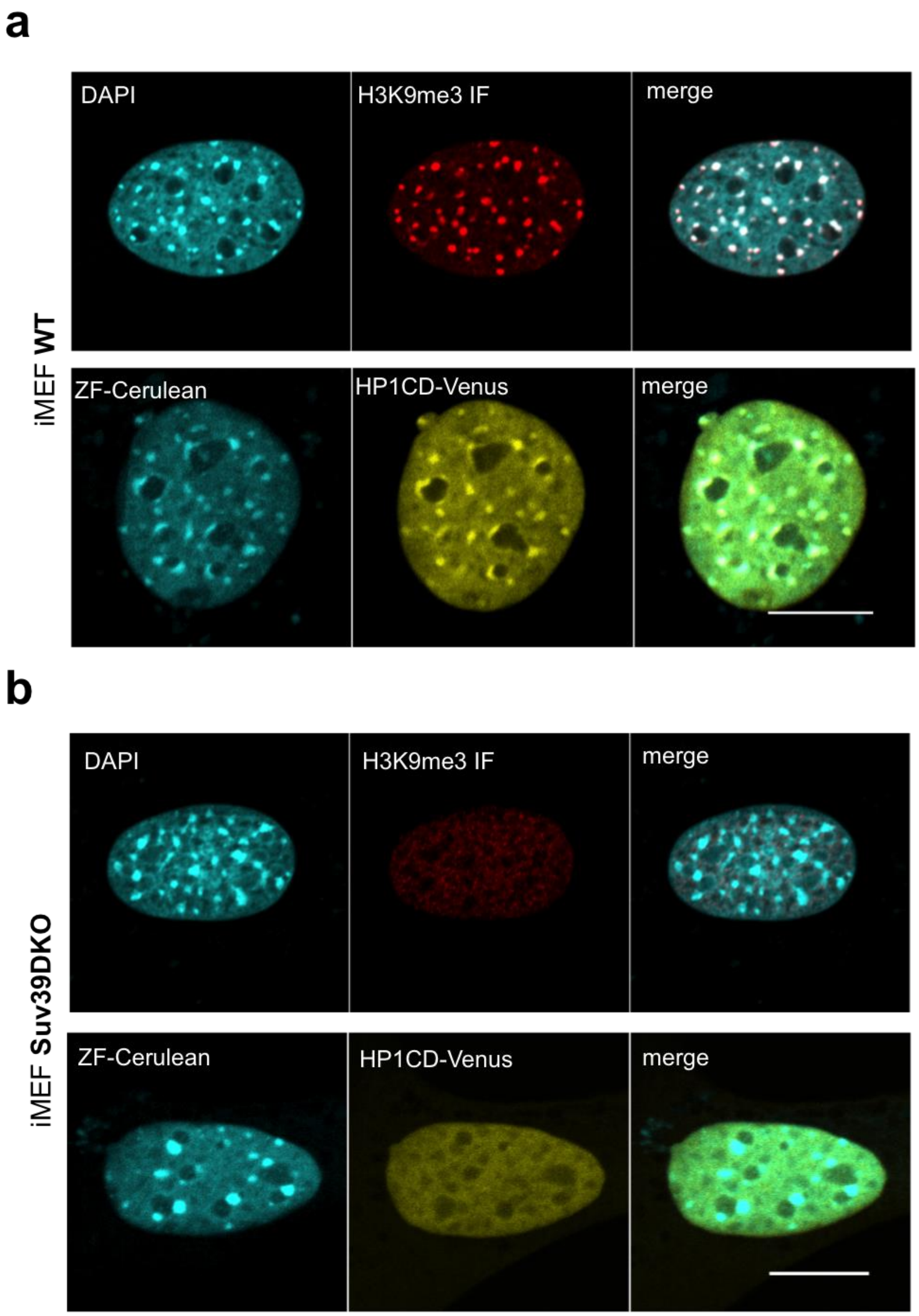

Supplementary Figure 21: Quality control of the H3K9me3 detector module used in BiAD sensor 4. Fluorescence microscopy images demonstrating that the ZF and the HP1CD modules (bottom panel) co-localize with the DAPI and H3K9me3 staining (top panel) respectively, observed in WT (**a**) and Suv39DKO (**b**) cells. This highlights the high DNA sequence and mark specificity of the two domains. This figure refers to Fig. 6.

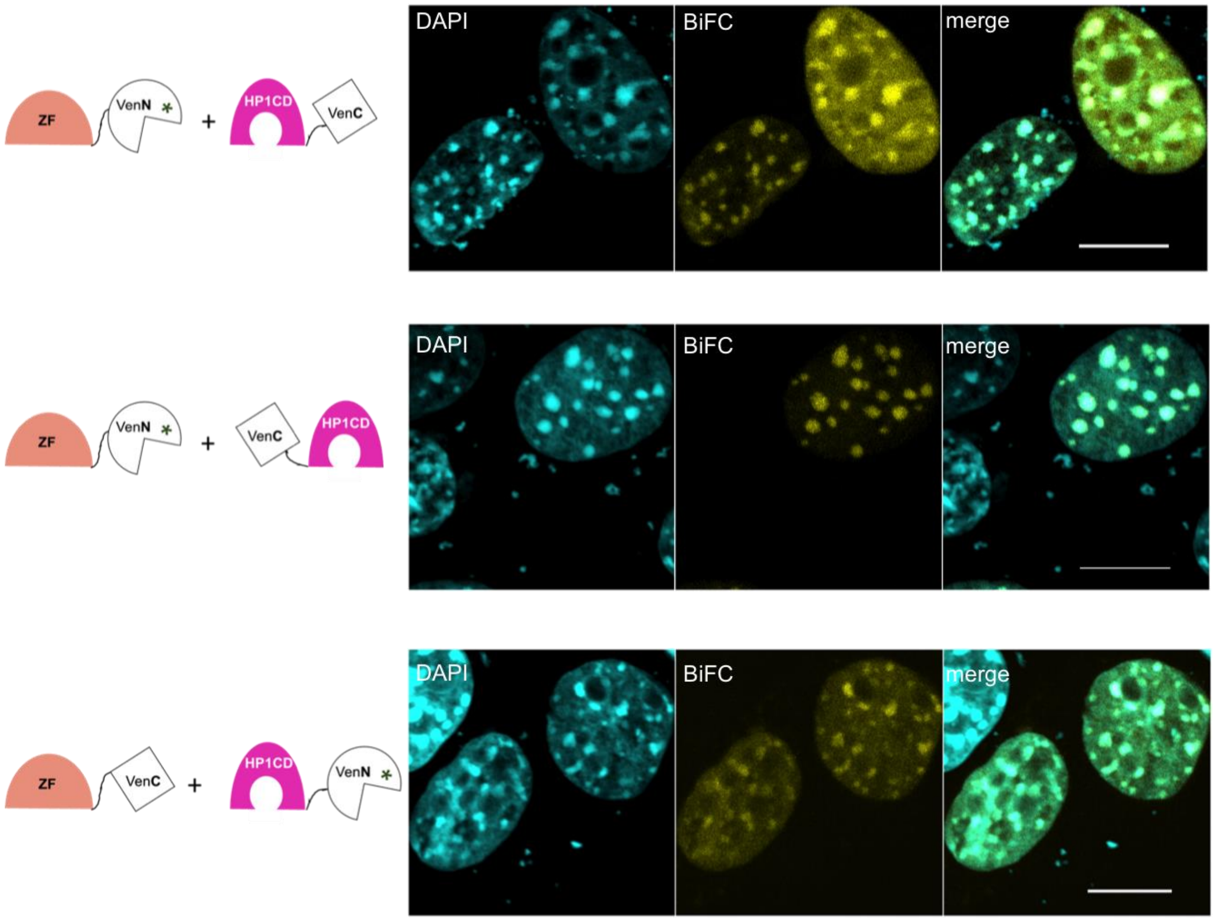

Supplementary Figure 22: Optimization of the BiFC yield of the BiAD sensor 4 by fluorophore permutations. Fluorescence microscopy images showing representative outcomes of the fluorophore rearrangement strategy employed to increase the sensitivity of the BiAD sensor 4. The tested combinations are schematically shown on the left of the corresponding image. Scale bar for all images is 10 µm. The cells were fixed 48h after transfection and the imaging and display setting of the images are identical between the 3 panels of the figure. The figure refers to Fig. 6.

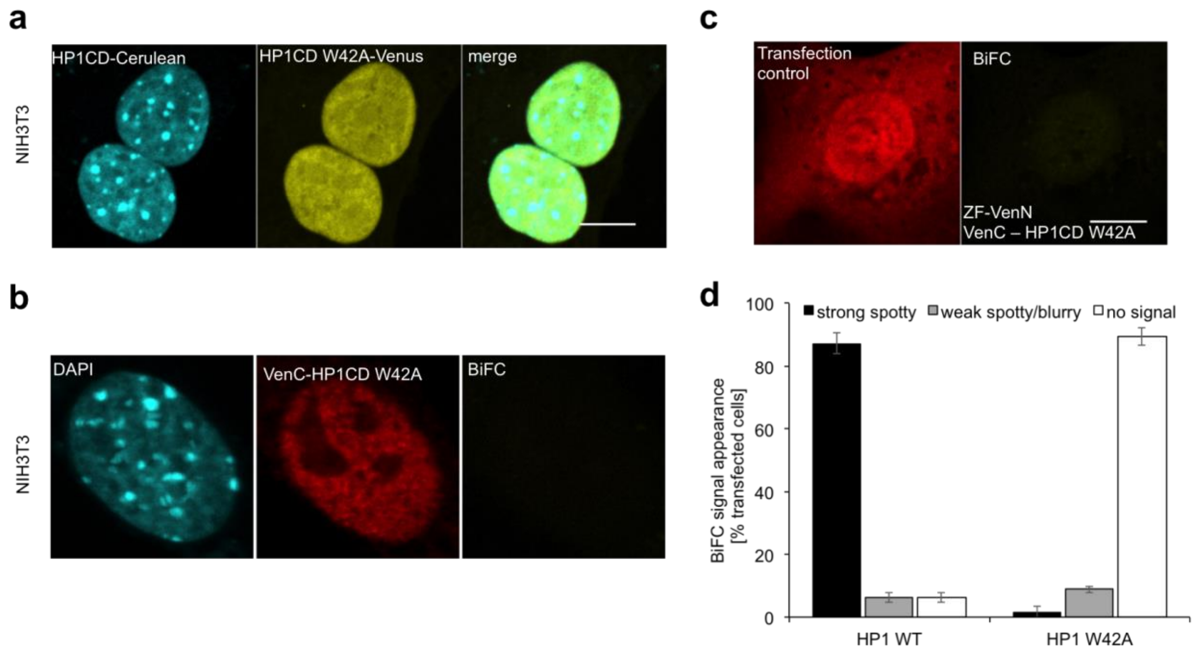

Supplementary Figure 23: Application of the HP1CD W42A variant to validate the H3K9me3 specificity of the BiAD sensor 4. W42A contains a mutations in the H3K9me3 trimethyllysine binding pocket that eliminates methyllysine binding (Nielsen et al., 2002). (**a**) Fluorescence microscopy images showing that sub-nuclear localization pattern of the HP1CD W42A domain mutant differs from the WT in NIH3T3. (**b**) Anti-FLAG immunofluorescence images demonstrating that like the full-fluorophore fusion, the VenC-fused HP1CD W42A no longer localizes to mouse chromocenters. (**c**) Representative fluorescence microscopy image documenting that high H3K9me3 specificity of the BiAD sensor 4. Upon implementation of the HP1CD W42A variant as a detector module, no BiFC signal was detectable. Cells were fixed 48h after transfection. A separate plasmid encoding for NLS-mRuby2 was used to identify transfected cells. Scale bar for all images is 10 µm. (**d**) Quantification of the number of cells with BiFC signal in the experiments representatively shown in panel c. Error bars represent s.e.m. for two biological replicates (for details cf. Supplementary Tables 4 and 6 and 7). This figure refers to Fig. 6.

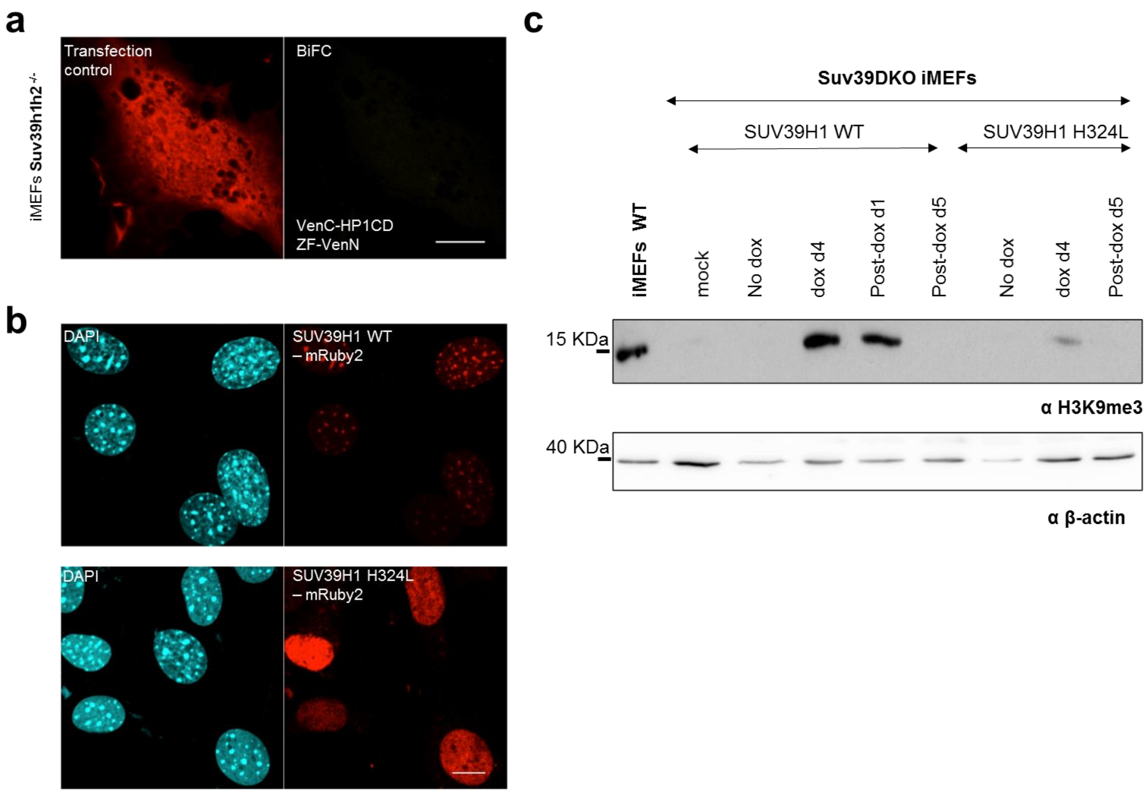

Supplementary Figure 24: Establishment of a model cell line for studying H3K9me3 dynamics. (**a**) Loss of BiFC signal formation in Suv39DKO cells with reduced H3K9me3 mark at pericentromeric repeats. (**b**) Fluorescence microscopy images documenting the successful and comparable protein expression of the WT SUV39H1 and inactive H324L SUV39H1 both fused to mRuby2 4 days post dox induction. The imaging and display setting of the images are identical between the 2 panels of the figure. (**c**) Western blot validation of the H3K9me3 recovery upon expression of the WT SUV39H1. The cell lysates were collected from original Suv39DKO cells (mock), before dox treatment (no dox), 4 days after the onset of dox treatment (dox d4), as well as 1 and 5 days after dox removal (post-dox d1 and d5). The catalytic activity of SUV39H1 was essential for H3K9me3 recovery. Equal volumes of lysates as what was used in the top blot were loaded on a separate SDS-PAGE and subjected to β-actin detection, as loading control. Scale bar for all images is 10 µm. The figure refers to Fig. 6.

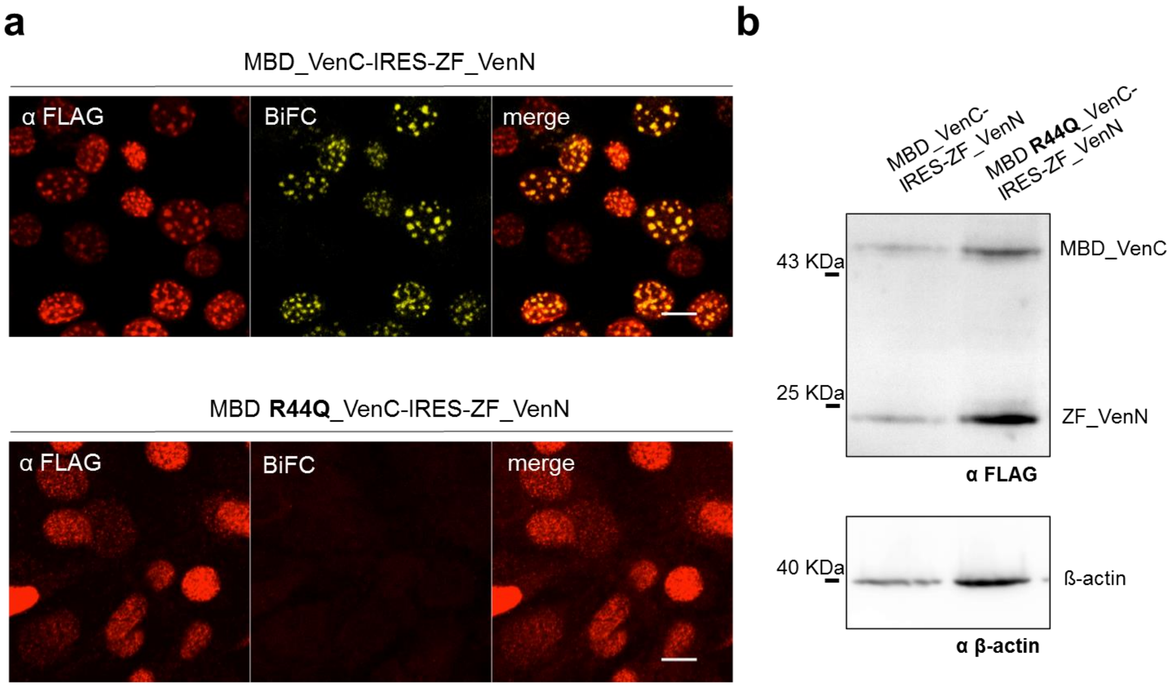

Supplementary Figure 25: Generation of cell lines stably expressing the BiAD 1 sensor. (**a**) Fluorescence microscopy images demonstrating that a BiFC signals are only detectable in the cell line expressing an intact MBD detector module (top panel) but not where the MBD R44Q was used (bottom panel). This shows that the 5mC specificity of the biosensor is preserved when its modules are constitutively expressed. Anti-FLAG immunofluorescence was used to detect the expression of the BiAD modules. Scale bar for all images is 10 µm. The imaging and display setting of the images are identical between the 2 panels of the figure. (**b**) Western blot validation of the expression of the FLAG-tagged anchor and detector modules in the cell lines shown in a. β-actin was used as a loading control. This figure refers to Fig. 7.

Supplementary figure 26. Sequences of the plasmids used in this study.

MBD-VenC

Annotated features:

3*FLAG

MBD of MBD1

Endogenous NLS

Linker

VenC

Full plasmid sequence:

GACGGATCGGGAGATCTCCCGATCCCCTATGGTCGACTCTCAGTACAATCTGCTCTGATGCCGCATAGTTAAGCCAGTATC
TGCTCCCTGCTTGTGTGTTGGAGGTCGCTGAGTAGTGCGCGAGCAAAATTTAAGCTACAACAAGGCAAGGCTTGACCGACA
ATTGCATGAAGAATCTGCTTAGGGTTAGGCGTTTTGCGCTGCTTCGCGATGTACGGGCCAGATATACGCGTTGACATTGAT
TATTGACTAGTTATTAATAGTAATCAATTACGGGGTCATTAGTTCATAGCCCATATATGGAGTTCCGCGTTACATAACTTACG
GTAAATGGCCCGCCTGGCTGACCGCCCAACGACCCCCGCCCATTGACGTCAATAATGACGTATGTTCCCATAGTAACGCC
AATAGGGACTTTCCATTGACGTCAATGGGTGGACTATTTACGGTAAACTGCCCACTTGGCAGTACATCAAGTGTATCATATG
CCAAGTACGCCCCCTATTGACGTCAATGACGGTAAATGGCCCGCCTGGCATTATGCCCAGTACATGACCTTATGGGACTTT
CCTACTTGGCAGTACATCTACGTATTAGTCATCGCTATTACCATGGTGATGCGGTTTTGGCAGTACATCAATGGGCGTGGAT
AGCGGTTTGACTCACGGGGATTTCCAAGTCTCCACCCCATTGACGTCAATGGGAGTTTGTTTTGGCACCAAAATCAACGGG
ACTTTCCAAAATGTCGTAACAACTCCGCCCCATTGACGCAAATGGGCGGTAGGCGTGTACGGTGGGAGGTCTATATAAGCA
GAGCTCTCTGGCTAACTAGAGAACCCACTGCTTACTGGCTTATCGAAATTAATACGACTCACTATAGGGAGACCCAAGCTTC
TCGGGCCCAATAATcATTTTATTTTGACTGATAGTGACCTGTTCGTTGCAACAAATTGATGAGCAATGCTTTTTTATAATGGAC
TATAAGGACCACGACGGAGACTACAAGGATCATGATATTGATTACAAAGACGATGACGATAAGATGGCTGAGGACTGGCTG
GACTGCCCGGCCCTGGGCCCTGGCTGGAAGCGCCGCGAAGTCTTTCGCAAGTCAGGGGCCACCTGTGGACGCTCAGACA
CCTATTACCAGAGCCCCACAGGAGACAGGATCCGAAGCAAAGTTGAGCTGACTCGATACCTGGGCCCTGCGTGTGATCTC
ACCCTCTTCGACTTCAAACAAGGCATCTTGTGCTATCCAGCCCCCAAGGCCCATCCCGTGGCGGTTGCCAGCAAGAAGCG
AAAGAAGCCTTCAAGGCCAGCCAAGACTCGGAAACGTCAGGTTGGACCCCAGAGTGGTGAGGTCAGGAAGGAGGCCCCG
ATCGATGGTGGCGGTGGCTCTGGAGGTGGTGGGTCCTCCGGAGACCCCAACGAGAAGCGCGATCACATGGTCCTGCTGG
AGTTCGTGACCGCCGCCGGGATCACTCTCGGCATGGACGAGCTGTACAAGTAATAATCTAGAGGGCCCTATTCTATAGTGT
CACCTAAATGCTAGAGCTCGCTGATCAGCCTCGACTGTGCCTTCTAGTTGCCAGCCATCTGTTGTTTGCCCCTCCCCCGTG
CCTTCCTTGACCCTGGAAGGTGCCACTCCCACTGTCCTTTCCTAATAAAATGAGGAAATTGCATCGCATTGTCTGAGTAGGT
GTCATTCTATTCTGGGGGGTGGGGTGGGGCAGGACAGCAAGGGGGAGGATTGGGAAGACAATAGCAGGCATGCTGGGGA
TGCGGTGGGCTCTATGGCTTCTGAGGCGGAAAGAACCAGCTGGGGCTCTAGGGGGTATCCCCACGCGCCCTGTAGCGGC
GCATTAAGCGCGGCGGGTGTGGTGGTTACGCGCAGCGTGACCGCTACACTTGCCAGCGCCCTAGCGCCCGCTCCTTTCG
CTTTCTTCCCTTCCTTTCTCGCCACGTTCGCCGGCTTTCCCCGTCAAGCTCTAAATCGGGGCATCCCTTTAGGGTTCCGATT
TAGTGCTTTACGGCACCTCGACCCCAAAAAACTTGATTAGGGTGATGGTTCACGTAGTGGGCCATCGCCCTGATAGACGGT
TTTTCGCCCTTTGACGTTGGAGTCCACGTTCTTTAATAGTGGACTCTTGTTCCAAACTGGAACAACACTCAACCCTATCTCG
GTCTATTCTTTTGATTTATAAGGGATTTTGGGGATTTCGGCCTATTGGTTAAAAAATGAGCTGATTTAACAAAAATTTAACGC
GAATTAATTCTGTGGAATGTGTGTCAGTTAGGGTGTGGAAAGTCCCCAGGCTCCCCAGGCAGGCAGAAGTATGCAAAGCAT
GCATCTCAATTAGTCAGCAACCAGGTGTGGAAAGTCCCCAGGCTCCCCAGCAGGCAGAAGTATGCAAAGCATGCATCTCAA
TTAGTCAGCAACCATAGTCCCGCCCCTAACTCCGCCCATCCCGCCCCTAACTCCGCCCAGTTCCGCCCATTCTCCGCCCCA
TGGCTGACTAATTTTTTTTATTTATGCAGAGGCCGAGGCCGCCTCTGCCTCTGAGCTATTCCAGAAGTAGTGAGGAGGCTTT
TTTGGAGGCCTAGGCTTTTGCAAAAAGCTCCCGGGAGCTTGTATATCCATTTTCGGATCTGATCAAGAGACAGGATGAGGA
TCGTTTCGCATGATTGAACAAGATGGATTGCACGCAGGTTCTCCGGCCGCTTGGGTGGAGAGGCTATTCGGCTATGACTG
GGCACAACAGACAATCGGCTGCTCTGATGCCGCCGTGTTCCGGCTGTCAGCGCAGGGGCGCCCGGTTCTTTTTGTCAAGA
CCGACCTGTCCGGTGCCCTGAATGAACTGCAGGACGAGGCAGCGCGGCTATCGTGGCTGGCCACGACGGGCGTTCCTTG
CGCAGCTGTGCTCGACGTTGTCACTGAAGCGGGAAGGGACTGGCTGCTATTGGGCGAAGTGCCGGGGCAGGATCTCCTG
TCATCTCACCTTGCTCCTGCCGAGAAAGTATCCATCATGGCTGATGCAATGCGGCGGCTGCATACGCTTGATCCGGCTACC
TGCCCATTCGACCACCAAGCGAAACATCGCATCGAGCGAGCACGTACTCGGATGGAAGCCGGTCTTGTCGATCAGGATGA
TCTGGACGAAGAGCATCAGGGGCTCGCGCCAGCCGAACTGTTCGCCAGGCTCAAGGCGCGCATGCCCGACGGCGAGGAT
CTCGTCGTGACCCATGGCGATGCCTGCTTGCCGAATATCATGGTGGAAAATGGCCGCTTTTCTGGATTCATCGACTGTGGC
CGGCTGGGTGTGGCGGACCGCTATCAGGACATAGCGTTGGCTACCCGTGATATTGCTGAAGAGCTTGGCGGCGAATGGG
CTGACCGCTTCCTCGTGCTTTACGGTATCGCCGCTCCCGATTCGCAGCGCATCGCCTTCTATCGCCTTCTTGACGAGTTCT
TCTGAGCGGGACTCTGGGGTTCGAAATGACCGACCAAGCGACGCCCAACCTGCCATCACGAGATTTCGATTCCACCGCCG
CCTTCTATGAAAGGTTGGGCTTCGGAATCGTTTTCCGGGACGCCGGCTGGATGATCCTCCAGCGCGGGGATCTCATGCTG
GAGTTCTTCGCCCACCCCAACTTGTTTATTGCAGCTTATAATGGTTACAAATAAAGCAATAGCATCACAAATTTCACAAATAA
AGCATTTTTTTCACTGCATTCTAGTTGTGGTTTGTCCAAACTCATCAATGTATCTTATCATGTCTGTATACCGTCGACCTCTAG
CTAGAGCTTGGCGTAATCATGGTCATAGCTGTTTCCTGTGTGAAATTGTTATCCGCTCACAATTCCACACAACATACGAGCC
GGAAGCATAAAGTGTAAAGCCTGGGGTGCCTAATGAGTGAGCTAACTCACATTAATTGCGTTGCGCTCACTGCCCGCTTTC
CAGTCGGGAAACCTGTCGTGCCAGCTGCATTAATGAATCGGCCAACGCGCGGGGAGAGGCGGTTTGCGTATTGGGCGCT
CTTCCGCTTCCTCGCTCACTGACTCGCTGCGCTCGGTCGTTCGGCTGCGGCGAGCGGTATCAGCTCACTCAAAGGCGGTA
ATACGGTTATCCACAGAATCAGGGGATAACGCAGGAAAGAACATGTGAGCAAAAGGCCAGCAAAAGGCCAGGAACCGTAA

AAAGGCCGCGTTGCTGGCGTTTTTCCATAGGCTCCGCCCCCCTGACGAGCATCACAAAAATCGACGCTCAAGTCAGAGGT
GGCGAAACCCGACAGGACTATAAAGATACCAGGCGTTTCCCCCTGGAAGCTCCCTCGTGCGCTCTCCTGTTCCGACCCTG
CCGCTTACCGGATACCTGTCCGCCTTTCTCCCTTCGGGAAGCGTGGCGCTTTCTCAATGCTCACGCTGTAGGTATCTCAGT
TCGGTGTAGGTCGTTCGCTCCAAGCTGGGCTGTGTGCACGAACCCCCCGTTCAGCCCGACCGCTGCGCCTTATCCGGTAA
CTATCGTCTTGAGTCCAACCCGGTAAGACACGACTTATCGCCACTGGCAGCAGCCACTGGTAACAGGATTAGCAGAGCGA
GGTATGTAGGCGGTGCTACAGAGTTCTTGAAGTGGTGGCCTAACTACGGCTACACTAGAAGGACAGTATTTGGTATCTGCG
CTCTGCTGAAGCCAGTTACCTTCGGAAAAAGAGTTGGTAGCTCTTGATCCGGCAAACAAACCACCGCTGGTAGCGGTGGTT
TTTTTGTTTGCAAGCAGCAGATTACGCGCAGAAAAAAAGGATCTCAAGAAGATCCTTTGATCTTTTCTACGGGGTCTGACGC
TCAGTGGAACGAAAACTCACGTTAAGGGATTTTGGTCATGAGATTATCAAAAAGGATCTTCACCTAGATCCTTTTAAATTAAA
AATGAAGTTTTAAATCAATCTAAAGTATATATGAGTAAACTTGGTCTGACAGTTACCAATGCTTAATCAGTGAGGCACCTATC
TCAGCGATCTGTCTATTTCGTTCATCCATAGTTGCCTGACTCCCCGTCGTGTAGATAACTACGATACGGGAGGGCTTACCAT
CTGGCCCCAGTGCTGCAATGATACCGCGAGACCCACGCTCACCGGCTCCAGATTTATCAGCAATAAACCAGCCAGCCGGA
AGGGCCGAGCGCAGAAGTGGTCCTGCAACTTTATCCGCCTCCATCCAGTCTATTAATTGTTGCCGGGAAGCTAGAGTAAGT
AGTTCGCCAGTTAATAGTTTGCGCAACGTTGTTGCCATTGCTACAGGCATCGTGGTGTCACGCTCGTCGTTTGGTATGGCT
TCATTCAGCTCCGGTTCCCAACGATCAAGGCGAGTTACATGATCCCCCATGTTGTGCAAAAAAGCGGTTAGCTCCTTCGGT
CCTCCGATCGTTGTCAGAAGTAAGTTGGCCGCAGTGTTATCACTCATGGTTATGGCAGCACTGCATAATTCTCTTACTGTCA
TGCCATCCGTAAGATGCTTTTCTGTGACTGGTGAGTACTCAACCAAGTCATTCTGAGAATAGTGTATGCGGCGACCGAGTT
GCTCTTGCCCGGCGTCAATACGGGATAATACCGCGCCACATAGCAGAACTTTAAAAGTGCTCATCATTGGAAAACGTTCTT
CGGGGCGAAAACTCTCAAGGATCTTACCGCTGTTGAGATCCAGTTCGATGTAACCCACTCGTGCACCCAACTGATCTTCAG
CATCTTTTACTTTCACCAGCGTTTCTGGGTGAGCAAAAACAGGAAGGCAAAATGCCGCAAAAAAGGGAATAAGGGCGACAC
GGAAATGTTGAATACTCATACTCTTCCTTTTTCAATATTATTGAAGCATTTATCAGGGTTATTGTCTCATGAGCGGATACATAT
TTGAATGTATTTAGAAAAATAAACAAATAGGGGTTCCGCGCACATTTCCCCGAAAAGTGCCACCTGACGTC

VenC-HP1CD

Annotated features:

3*FLAG

VenC

Linker

SV40 NLS

Chromodomain of HP1

Full plasmid sequence:

TAGTTATTAATAGTAATCAATTACGGGGTCATTAGTTCATAGCCCATATATGGAGTTCCGCGTTACATAACTTACGGTAAATG
GCCCGCCTGGCTGACCGCCCAACGACCCCCGCCCATTGACGTCAATAATGACGTATGTTCCCATAGTAACGCCAATAGGG
ACTTTCCATTGACGTCAATGGGTGGAGTATTTACGGTAAACTGCCCACTTGGCAGTACATCAAGTGTATCATATGCCAAGTA
CGCCCCCTATTGACGTCAATGACGGTAAATGGCCCGCCTGGCATTATGCCCAGTACATGACCTTATGGGACTTTCCTACTT
GGCAGTACATCTACGTATTAGTCATCGCTATTACCATGGTGATGCGGTTTTGGCAGTACATCAATGGGCGTGGATAGCGGT
TTGACTCACGGGGATTTCCAAGTCTCCACCCCATTGACGTCAATGGGAGTTTGTTTTGGCACCAAAATCAACGGGACTTTCC
AAAATGTCGTAACAACTCCGCCCCATTGACGCAAATGGGCGGTAGGCGTGTACGGTGGGAGGTCTATATAAGCAGAGCTG
GTTTAGTGAACCGTCAGATCCGCTAGCGCTACCGGTCGCCACCATGGACTATAAGGACCACGACGGAGACTACAAGGATC
ATGATATTGATTACAAAGACGATGACGATAAGGACCCCAACGAGAAGCGCGATCACATGGTCCTGCTGGAGTTCGTGACCG
CCGCCGGGATCACTCTCGGCATGGACGAGCTGTACAAGATCGATGGTGGCGGTGGCTCTGGAGGTGGTGGGTCCTCCGG
ACCAAAGAAGAAGCGTAAGGTAGAGGAAGAGGAATATGTGGTGGAAAAAGTTCTTGATCGGCGAGTTGTCAAGGGCAAGG
TGGAATATCTTCTAAAGTGGAAGGGTTTCTCAGATGAGGACAACACTTGGGAGCCAGAAGAGAATCTGGATTGCCCTGACC
TTATTGCTGAGTTTCTACAGTCACAGAAAACAGCACATGAGTAATCCGGACTCAGATCTCGAGCTCAAGCTTCGAATTCTGC
AGTCGACGGTACCGCGGGCCCGGGATCCACCGGATCTAGATAACTGATCATAATCAGCCATACCACATTTGTAGAGGTTTT
ACTTGCTTTAAAAAACCTCCCACACCTCCCCCTGAACCTGAAACATAAAATGAATGCAATTGTTGTTGTTAACTTGTTTATTG
CAGCTTATAATGGTTACAAATAAAGCAATAGCATCACAAATTTCACAAATAAAGCATTTTTTTCACTGCATTCTAGTTGTGGTT
TGTCCAAACTCATCAATGTATCTTAACGCGTAAATTGTAAGCGTTAATATTTTGTTAAAATTCGCGTTAAATTTTTGTTAAATC
AGCTCATTTTTTAACCAATAGGCCGAAATCGGCAAAATCCCTTATAAATCAAAAGAATAGACCGAGATAGGGTTGAGTGTTG
TTCCAGTTTGGAACAAGAGTCCACTATTAAAGAACGTGGACTCCAACGTCAAAGGGCGAAAAACCGTCTATCAGGGCGATG
GCCCACTACGTGAACCATCACCCTAATCAAGTTTTTTGGGGTCGAGGTGCCGTAAAGCACTAAATCGGAACCCTAAAGGGA
GCCCCCGATTTAGAGCTTGACGGGGAAAGCCGGCGAACGTGGCGAGAAAGGAAGGGAAGAAAGCGAAAGGAGCGGGCG
CTAGGGCGCTGGCAAGTGTAGCGGTCACGCTGCGCGTAACCACCACACCCGCCGCGCTTAATGCGCCGCTACAGGGCGC
GTCAGGTGGCACTTTTCGGGGAAATGTGCGCGGAACCCCTATTTGTTTATTTTTCTAAATACATTCAAATATGTATCCGCTCA
TGAGACAATAACCCTGATAAATGCTTCAATAATATTGAAAAAGGAAGAGTCCTGAGGCGGAAAGAACCAGCTGTGGAATGT
GTGTCAGTTAGGGTGTGGAAAGTCCCCAGGCTCCCCAGCAGGCAGAAGTATGCAAAGCATGCATCTCAATTAGTCAGCAA
CCAGGTGTGGAAAGTCCCCAGGCTCCCCAGCAGGCAGAAGTATGCAAAGCATGCATCTCAATTAGTCAGCAACCATAGTC
CCGCCCCTAACTCCGCCCATCCCGCCCCTAACTCCGCCCAGTTCCGCCCATTCTCCGCCCCATGGCTGACTAATTTTTTTT
ATTTATGCAGAGGCCGAGGCCGCCTCGGCCTCTGAGCTATTCCAGAAGTAGTGAGGAGGCTTTTTTGGAGGCCTAGGCTT
TTGCAAAGATCGATCAAGAGACAGGATGAGGATCGTTTCGCATGATTGAACAAGATGGATTGCACGCAGGTTCTCCGGCCG

CTTGGGTGGAGAGGCTATTCGGCTATGACTGGGCACAACAGACAATCGGCTGCTCTGATGCCGCCGTGTTCCGGCTGTCA
GCGCAGGGGCGCCCGGTTCTTTTTGTCAAGACCGACCTGTCCGGTGCCCTGAATGAACTGCAAGACGAGGCAGCGCGGC
TATCGTGGCTGGCCACGACGGGCGTTCCTTGCGCAGCTGTGCTCGACGTTGTCACTGAAGCGGGAAGGGACTGGCTGCT
ATTGGGCGAAGTGCCGGGGCAGGATCTCCTGTCATCTCACCTTGCTCCTGCCGAGAAAGTATCCATCATGGCTGATGCAAT
GCGGCGGCTGCATACGCTTGATCCGGCTACCTGCCCATTCGACCACCAAGCGAAACATCGCATCGAGCGAGCACGTACTC
GGATGGAAGCCGGTCTTGTCGATCAGGATGATCTGGACGAAGAGCATCAGGGGCTCGCGCCAGCCGAACTGTTCGCCAG
GCTCAAGGCGAGCATGCCCGACGGCGAGGATCTCGTCGTGACCCATGGCGATGCCTGCTTGCCGAATATCATGGTGGAAA
ATGGCCGCTTTTCTGGATTCATCGACTGTGGCCGGCTGGGTGTGGCGGACCGCTATCAGGACATAGCGTTGGCTACCCGT
GATATTGCTGAAGAGCTTGGCGGCGAATGGGCTGACCGCTTCCTCGTGCTTTACGGTATCGCCGCTCCCGATTCGCAGCG
CATCGCCTTCTATCGCCTTCTTGACGAGTTCTTCTGAGCGGGACTCTGGGGTTCGAAATGACCGACCAAGCGACGCCCAAC
CTGCCATCACGAGATTTCGATTCCACCGCCGCCTTCTATGAAAGGTTGGGCTTCGGAATCGTTTTCCGGGACGCCGGCTG
GATGATCCTCCAGCGCGGGGATCTCATGCTGGAGTTCTTCGCCCACCCTAGGGGGAGGCTAACTGAAACACGGAAGGAGA
CAATACCGGAAGGAACCCGCGCTATGACGGCAATAAAAAGACAGAATAAAACGCACGGTGTTGGGTCGTTTGTTCATAAAC
GCGGGGTTCGGTCCCAGGGCTGGCACTCTGTCGATACCCCACCGAGACCCCATTGGGGCCAATACGCCCGCGTTTCTTC
CTTTTCCCCACCCCACCCCCCAAGTTCGGGTGAAGGCCCAGGGCTCGCAGCCAACGTCGGGGCGGCAGGCCCTGCCATA
GCCTCAGGTTACTCATATATACTTTAGATTGATTTAAAACTTCATTTTTAATTTAAAAGGATCTAGGTGAAGATCCTTTTTGAT
AATCTCATGACCAAAATCCCTTAACGTGAGTTTTCGTTCCACTGAGCGTCAGACCCCGTAGAAAAGATCAAAGGATCTTCTT
GAGATCCTTTTTTTCTGCGCGTAATCTGCTGCTTGCAAACAAAAAAACCACCGCTACCAGCGGTGGTTTGTTTGCCGGATCA
AGAGCTACCAACTCTTTTTCCGAAGGTAACTGGCTTCAGCAGAGCGCAGATACCAAATACTGTCCTTCTAGTGTAGCCGTA
GTTAGGCCACCACTTCAAGAACTCTGTAGCACCGCCTACATACCTCGCTCTGCTAATCCTGTTACCAGTGGCTGCTGCCAG
TGGCGATAAGTCGTGTCTTACCGGGTTGGACTCAAGACGATAGTTACCGGATAAGGCGCAGCGGTCGGGCTGAACGGGG
GGTTCGTGCACACAGCCCAGCTTGGAGCGAACGACCTACACCGAACTGAGATACCTACAGCGTGAGCTATGAGAAAGCGC
CACGCTTCCCGAAGGGAGAAAGGCGGACAGGTATCCGGTAAGCGGCAGGGTCGGAACAGGAGAGCGCACGAGGGAGCT
TCCAGGGGGAAACGCCTGGTATCTTTATAGTCCTGTCGGGTTTCGCCACCTCTGACTTGAGCGTCGATTTTTGTGATGCTC
GTCAGGGGGGCGGAGCCTATGGAAAAACGCCAGCAACGCGGCCTTTTTACGGTTCCTGGCCTTTTGCTGGCCTTTTGCTC
ACATGTTCTTTCCTGCGTTATCCCCTGATTCTGTGGATAACCGTATTACCGCCATGCAT

ZF-VenN

Annotated features:

3*FLAG

SV40 NLS

ZF

Linker

VenN

Full plasmid sequence:

TAGTTATTAATAGTAATCAATTACGGGGTCATTAGTTCATAGCCCATATATGGAGTTCCGCGTTACATAACTTACGGTAAATG
GCCCGCCTGGCTGACCGCCCAACGACCCCCGCCCATTGACGTCAATAATGACGTATGTTCCCATAGTAACGCCAATAGGG
ACTTTCCATTGACGTCAATGGGTGGAGTATTTACGGTAAACTGCCCACTTGGCAGTACATCAAGTGTATCATATGCCAAGTA
CGCCCCCTATTGACGTCAATGACGGTAAATGGCCCGCCTGGCATTATGCCCAGTACATGACCTTATGGGACTTTCCTACTT
GGCAGTACATCTACGTATTAGTCATCGCTATTACCATGGTGATGCGGTTTTGGCAGTACATCAATGGGCGTGGATAGCGGT
TTGACTCACGGGGATTTCCAAGTCTCCACCCCATTGACGTCAATGGGAGTTTGTTTTGGCACCAAAATCAACGGGACTTTCC
AAAATGTCGTAACAACTCCGCCCCATTGACGCAAATGGGCGGTAGGCGTGTACGGTGGGAGGTCTATATAAGCAGAGCTG
GTTTAGTGAACCGTCAGATCCGCTAGCGCTACCGGTCGCCACCATGGACTATAAGGACCACGACGGAGACTACAAGGATC
ATGATATTGATTACAAAGACGATGACGATAAGCCTAAGAAGAAGCGTAAGGTAGGCGAGAAACCGTACGCTTGCCCTGAAT
GCGGAAAAAGCTTCTCTCAGTCCAGCAACCTCGTGAGGCATCAGAGGACCCATACCGGCGAGAAGCCCTACGCTTGCCCG
GAGTGTGGCAAGTCATTCTCCAGGAGCGACAATTTGGTTAGGCATCAACGCACACATACCGGCGAGAAACCGTACGCGTG
CCCTGAATGCGGTAAAAGCTTCTCTGACCCAGGTCACCTCGTCCGCCATCAGAGGACCCATACCACTGGATCGTCACAAAC
CTCGGGTTCATCCACGTCGGAAGGCCAGGAGGGCACGGTGAGCAAGGGCGAGGAGCTGTTCACCGGGGTGGTGCCCAT
CCTGGTCGAGCTGGACGGCGACGTAAACGGCCACAAGTTCAGCGTGTCCGGCGAGGGCGAGGGCGATGCCACCTACGG
CAAGCTGACCCTGAAGCTCATCTGCACCACCGGCAAGCTGCCCGTGCCCTGGCCCACCCTCGTGACCACCCTCGGCTAC
GGCCTGCAGTGCTTCGCCCGCTACCCCGACCACATGAAGCAGCACGACTTCTTCAAGTCCGCCATGCCCGAAGGCTACGT
CCAGGAGCGCACCATCTTCTTCAAGGACGACGGCAACTACAAGACCCGCGCCGAGGTGAAGTTCGAGGGCGACACCCTG
GTGAACCGCATCGAGCTGAAGGGCATCGACTTCAAGGAGGACGGCAACATCCTGGGGCACAAGCTGGAGTACAACTACAA
CAGCCACAACGTCTATATCACCGCCGACAAGCAGAAGAACGGCATCAAGGCCAACTTCAAGATCCGCCACAACATCGAGG
ACGGCGGCGTGCAGCTCGCCGACCACTACCAGCAGAACACCCCCATCGGCGACGGCCCCGTGCTGCTGCCCGACAACCA
CTACCTGAGCTACCAGTCCAAGCTGAGCAAAGACTAATCCGGACTCAGATCTCGAGCTCAAGCTTCGAATTCTGCAGTCGA
CGGTACCGCGGGCCCGGGATCCACCGGATCTAGATAACTGATCATAATCAGCCATACCACATTTGTAGAGGTTTTACTTGC
TTTAAAAAACCTCCCACACCTCCCCCTGAACCTGAAACATAAAATGAATGCAATTGTTGTTGTTAACTTGTTTATTGCAGCTT
ATAATGGTTACAAATAAAGCAATAGCATCACAAATTTCACAAATAAAGCATTTTTTTCACTGCATTCTAGTTGTGGTTTGTCCA
AACTCATCAATGTATCTTAAGGCGTAAATTGTAAGCGTTAATATTTTGTTAAAATTCGCGTTAAATTTTTGTTAAATCAGCTCA
TTTTTTAACCAATAGGCCGAAATCGGCAAAATCCCTTATAAATCAAAAGAATAGACCGAGATAGGGTTGAGTGTTGTTCCAG
TTTGGAACAAGAGTCCACTATTAAAGAACGTGGACTCCAACGTCAAAGGGCGAAAAACCGTCTATCAGGGCGATGGCCCAC

TACGTGAACCATCACCCTAATCAAGTTTTTTGGGGTCGAGGTGCCGTAAAGCACTAAATCGGAACCCTAAAGGGAGCCCCC
GATTTAGAGCTTGACGGGGAAAGCCGGCGAACGTGGCGAGAAAGGAAGGGAAGAAAGCGAAAGGAGCGGGCGCTAGGG
CGCTGGCAAGTGTAGCGGTCACGCTGCGCGTAACCACCACACCCGCCGCGCTTAATGCGCCGCTACAGGGCGCGTCAGG
TGGCACTTTTCGGGGAAATGTGCGCGGAACCCCTATTTGTTTATTTTTCTAAATACATTCAAATATGTATCCGCTCATGAGAC
AATAACCCTGATAAATGCTTCAATAATATTGAAAAAGGAAGAGTCCTGAGGCGGAAAGAACCAGCTGTGGAATGTGTGTCA
GTTAGGGTGTGGAAAGTCCCCAGGCTCCCCAGCAGGCAGAAGTATGCAAAGCATGCATCTCAATTAGTCAGCAACCAGGT
GTGGAAAGTCCCCAGGCTCCCCAGCAGGCAGAAGTATGCAAAGCATGCATCTCAATTAGTCAGCAACCATAGTCCCGCCC
CTAACTCCGCCCATCCCGCCCCTAACTCCGCCCAGTTCCGCCCATTCTCCGCCCCATGGCTGACTAATTTTTTTTATTTATG
CAGAGGCCGAGGCCGCCTCGGCCTCTGAGCTATTCCAGAAGTAGTGAGGAGGCTTTTTTGGAGGCCTAGGCTTTTGCAAA
GATCGATCAAGAGACAGGATGAGGATCGTTTCGCATGATTGAACAAGATGGATTGCACGCAGGTTCTCCGGCCGCTTGGG
TGGAGAGGCTATTCGGCTATGACTGGGCACAACAGACAATCGGCTGCTCTGATGCCGCCGTGTTCCGGCTGTCAGCGCAG
GGGCGCCCGGTTCTTTTTGTCAAGACCGACCTGTCCGGTGCCCTGAATGAACTGCAAGACGAGGCAGCGCGGCTATCGTG
GCTGGCCACGACGGGCGTTCCTTGCGCAGCTGTGCTCGACGTTGTCACTGAAGCGGGAAGGGACTGGCTGCTATTGGGC
GAAGTGCCGGGGCAGGATCTCCTGTCATCTCACCTTGCTCCTGCCGAGAAAGTATCCATCATGGCTGATGCAATGCGGCG
GCTGCATACGCTTGATCCGGCTACCTGCCCATTCGACCACCAAGCGAAACATCGCATCGAGCGAGCACGTACTCGGATGG
AAGCCGGTCTTGTCGATCAGGATGATCTGGACGAAGAGCATCAGGGGCTCGCGCCAGCCGAACTGTTCGCCAGGCTCAA
GGCGAGCATGCCCGACGGCGAGGATCTCGTCGTGACCCATGGCGATGCCTGCTTGCCGAATATCATGGTGGAAAATGGC
CGCTTTTCTGGATTCATCGACTGTGGCCGGCTGGGTGTGGCGGACCGCTATCAGGACATAGCGTTGGCTACCCGTGATAT
TGCTGAAGAGCTTGGCGGCGAATGGGCTGACCGCTTCCTCGTGCTTTACGGTATCGCCGCTCCCGATTCGCAGCGCATCG
CCTTCTATCGCCTTCTTGACGAGTTCTTCTGAGCGGGACTCTGGGGTTCGAAATGACCGACCAAGCGACGCCCAACCTGC
CATCACGAGATTTCGATTCCACCGCCGCCTTCTATGAAAGGTTGGGCTTCGGAATCGTTTTCCGGGACGCCGGCTGGATGA
TCCTCCAGCGCGGGGATCTCATGCTGGAGTTCTTCGCCCACCCTAGGGGGAGGCTAACTGAAACACGGAAGGAGACAATA
CCGGAAGGAACCCGCGCTATGACGGCAATAAAAAGACAGAATAAAACGCACGGTGTTGGGTCGTTTGTTCATAAACGCGG
GGTTCGGTCCCAGGGCTGGCACTCTGTCGATACCCCACCGAGACCCCATTGGGGCCAATACGCCCGCGTTTCTTCCTTTT
CCCCACCCCACCCCCCAAGTTCGGGTGAAGGCCCAGGGCTCGCAGCCAACGTCGGGGCGGCAGGCCCTGCCATAGCCT
CAGGTTACTCATATATACTTTAGATTGATTTAAAACTTCATTTTTAATTTAAAAGGATCTAGGTGAAGATCCTTTTTGATAATCT
CATGACCAAAATCCCTTAACGTGAGTTTTCGTTCCACTGAGCGTCAGACCCCGTAGAAAAGATCAAAGGATCTTCTTGAGAT
CCTTTTTTTCTGCGCGTAATCTGCTGCTTGCAAACAAAAAAACCACCGCTACCAGCGGTGGTTTGTTTGCCGGATCAAGAGC
TACCAACTCTTTTTCCGAAGGTAACTGGCTTCAGCAGAGCGCAGATACCAAATACTGTCCTTCTAGTGTAGCCGTAGTTAGG
CCACCACTTCAAGAACTCTGTAGCACCGCCTACATACCTCGCTCTGCTAATCCTGTTACCAGTGGCTGCTGCCAGTGGCGA
TAAGTCGTGTCTTACCGGGTTGGACTCAAGACGATAGTTACCGGATAAGGCGCAGCGGTCGGGCTGAACGGGGGGTTCGT
GCACACAGCCCAGCTTGGAGCGAACGACCTACACCGAACTGAGATACCTACAGCGTGAGCTATGAGAAAGCGCCACGCTT
CCCGAAGGGAGAAAGGCGGACAGGTATCCGGTAAGCGGCAGGGTCGGAACAGGAGAGCGCACGAGGGAGCTTCCAGGG
GGAAACGCCTGGTATCTTTATAGTCCTGTCGGGTTTCGCCACCTCTGACTTGAGCGTCGATTTTTGTGATGCTCGTCAGGG
GGGCGGAGCCTATGGAAAAACGCCAGCAACGCGGCCTTTTTACGGTTCCTGGCCTTTTGCTGGCCTTTTGCTCACATGTTC
TTTCCTGCGTTATCCCCTGATTCTGTGGATAACCGTATTACCGCCATGCAT

TALE-VenN

Annotated features:

3*FLAG

SV40 NLS

TALE

Linker

VenN

Full plasmid sequence:

ACGGATCGGGAGATCTCCCGATCCCCTATGGTGCACTCTCAGTACAATCTGCTCTGATGCCGCATAGTTAAGCCAGTATCT
GCTCCCTGCTTGTGTGTTGGAGGTCGCTGAGTAGTGCGCGAGCAAAATTTAAGCTACAACAAGGCAAGGCTTGACCGACA
ATTCCATGAAGAATCTGCTTAGGGTTAGGCGTTTTGCGCTGCTTCGCGATGTACGGGCCAGATATACGCGTTGACATTGAT
TATTGACTAGTTATTAATAGTAATCAATTACGGGGTCATTAGTTCATAGCCCATATATGGAGTTCCGCGTTACATAACTTACG
GTAAATGGCCCGCCTGGCTGACCGCCCAACGACCCCCGCCCATTGACGTCAATAATGACGTATGTTCCCATAGTAACGCC
AATAGGGACTTTCCATTGACGTCAATGGGTGGAGTATTTACGGTAAACTGCCCACTTGGCAGTACATCAAGTGTATCATATG
CCAAGTACGCCCCCTATTGACGTCAATGACGGTAAATGGCCCGCCTGGCATTATGCCCAGTACATGACCTTATGGGACTTT
CCTACTTGGCAGTACATCTACGTATTAGTCATCGCTATTACCATGGTGATGCGGTTTTGGCAGTACATCAATGGGCGTGGAT
AGCGGTTTGACTCACGGGGATTTCCAAGTCTCCACCCCATTGACGTCAATGGGAGTTTGTTTTGGCACCAAAATCAACGGG
ACTTTCCAAAATGTCGTAACAACTCCGCCCCATTGACGCAAATGGGCGGTAGGCGTGTACGGTGGGAGGTCTATATAAGCA
GAGCTCTCCCTATCAGTGATAGAGATCTCCCTATCAGTGATAGAGATCGTCGACGAGCTCGTTTAGTGAACCGTCAGATCG
CCTGGAGACGCCATCCACGCTGTTTTGACCTCCATAGAAGACACCGGGACCGATCCAGCCTGGCGCGCCGGTTCTGGATC
CGGTTCTCAATTGGGTTCTACCGGTTAGGGGCCACCATGGACTATAAGGACCACGACGGAGACTACAAGGATCATGATATT
GATTACAAAGACGATGACGATAAGATGGCCCCAAAGAAGAAGCGGAAGGTCGGTATCCACGGAGTCCCAGCAGCCGTAGA
TTTGAGAACTTTGGGATATTCACAGCAGCAGCAGGAAAAGATCAAGCCCAAAGTGAGGTCGACAGTCGCGCAGCATCACG
AAGCGCTGGTGGGTCATGGGTTTACACATGCCCACATCGTAGCCTTGTCGCAGCACCCTGCAGCCCTTGGCACGGTCGCC
GTCAAGTACCAGGACATGATTGCGGCGTTGCCGGAAGCCACACATGAGGCGATCGTCGGTGTGGGGAAACAGTGGAGCG

GAGCCCGAGCGCTTGAGGCCCTGTTGACGGTCGCGGGAGAGCTGAGAGGGCCTCCCCTTCAGCTGGACACGGCCAGTT
GCTGAAGATCGCGAAGCGGGGAGGAGTCACGGCGGTCGAGGCGGTGCACGCGTGGCGCAATGCGCTCACGGGAGCACC
CCTCAACCTGACCCCAGAGCAGGTCGTGGCAATTGCGAGCAACATCGGGGGAAAGCAGGCACTCGAAACCGTCCAGAGG
TTGCTGCCTGTGCTGTGCCAAGCGCACGGACTTACGCCAGAGCAGGTCGTGGCAATTGCGAGCAACCACGGGGGAAAGC
AGGCACTCGAAACCGTCCAGAGGTTGCTGCCTGTGCTGTGCCAAGCGCACGGACTAACCCCAGAGCAGGTCGTGGCAATT
GCGAGCAACATCGGGGGAAAGCAGGCACTCGAAACCGTCCAGAGGTTGCTGCCTGTGCTGTGCCAAGCGCACGGGTTGA
CCCCAGAGCAGGTCGTGGCAATTGCGAGCCATGACGGGGGAAAGCAGGCACTCGAAACCGTCCAGAGGTTGCTGCCTGT
GCTGTGCCAAGCGCACGGCCTGACCCCAGAGCAGGTCGTGGCAATTGCGAGCAACATCGGGGGAAAGCAGGCACTCGAA
ACCGTCCAGAGGTTGCTGCCTGTGCTGTGCCAAGCGCACGGACTGACACCAGAGCAGGTCGTGGCAATTGCGAGCAACC
ACGGGGGAAAGCAGGCACTCGAAACCGTCCAGAGGTTGCTGCCTGTGCTGTGCCAAGCGCACGGACTTACACCCGAACA
AGTCGTGGCAATTGCGAGCAACATCGGGGGAAAGCAGGCACTCGAAACCGTCCAGAGGTTGCTGCCTGTGCTGTGCCAA
GCGCACGGACTTACGCCAGAGCAGGTCGTGGCAATTGCGAGCAACATCGGGGGAAAGCAGGCACTCGAAACCGTCCAGA
GGTTGCTGCCTGTGCTGTGCCAAGCGCACGGACTAACCCCAGAGCAGGTCGTGGCAATTGCGAGCAACCACGGGGGAAA
GCAGGCACTCGAAACCGTCCAGAGGTTGCTGCCTGTGCTGTGCCAAGCGCACGGGTTGACCCCAGAGCAGGTCGTGGCA
ATTGCGAGCCATGACGGGGGAAAGCAGGCACTCGAAACCGTCCAGAGGTTGCTGCCTGTGCTGTGCCAAGCGCACGGCC
TGACCCCAGAGCAGGTCGTGGCAATTGCGAGCAACATCGGGGGAAAGCAGGCACTCGAAACCGTCCAGAGGTTGCTGCC
TGTGCTGTGCCAAGCGCACGGACTGACACCAGAGCAGGTCGTGGCAATTGCGAGCAACGGAGGGGGAAAGCAGGCACTC
GAAACCGTCCAGAGGTTGCTGCCTGTGCTGTGCCAAGCGCACGGCCTCACCCCAGAGCAGGTCGTGGCAATTGCGAGCA
ACGGAGGGGGAAAGCAGGCACTCGAAACCGTCCAGAGGTTGCTGCCTGTGCTGTGCCAAGCGCACGGACTTACGCCAGA
GCAGGTCGTGGCAATTGCGAGCCATGACGGGGGAAAGCAGGCACTCGAAACCGTCCAGAGGTTGCTGCCTGTGCTGTGC
CAAGCGCACGGACTAACCCCAGAGCAGGTCGTGGCAATTGCGAGCAACGGAGGGGGAAAGCAGGCACTCGAAACCGTCC
AGAGGTTGCTGCCTGTGCTGTGCCAAGCGCACGGGTTGACCCCAGAGCAGGTCGTGGCAATTGCGAGCCATGACGGGGG
AAAGCAGGCACTCGAAACCGTCCAGAGGTTGCTGCCTGTGCTGTGCCAAGCGCACGGCCTGACCCCAGAGCAGGTCGTG
GCAATTGCGAGCAACATCGGGGGAAAGCAGGCACTCGAAACCGTCCAGAGGTTGCTGCCTGTGCTGTGCCAAGCGCACG
GACTGACACCAGAGCAGGTCGTGGCAATTGCGAGCAACCACGGGGGAAAGCAGGCACTCGAAACCGTCCAGAGGTTGCT
GCCTGTGCTGTGCCAAGCGCACGGACTCACGCCTGAGCAGGTAGTGGCTATTGCATCCAACATCGGGGGCAGACCCGCA
CTGGAGTCAATCGTGGCCCAGCTTTCGAGGCCGGACCCCGCGCTGGCCGCACTCACTAATGATCATCTTGTAGCGCTGGC
CTGCCTCGGCGGACGACCCGCCTTGGATGCGGTGAAGAAGGGGCTCCCGCACGCGCCTGCATTGATTAAGCGGACCAAC
AGAAGGATTCCCGAGAGGACATCACATCGAGTGGCAGGCCTGCAGGGAAGTGGAAGTGTGAGCAAGGGCGAGGAGCTGT
TCACCGGGGTGGTGCCCATCCTGGTCGAGCTGGACGGCGACGTAAACGGCCACAAGTTCAGCGTGTCCGGCGAGGGCGA
GGGCGATGCCACCTACGGCAAGCTGACCCTGAAGCTCATCTGCACCACCGGCAAGCTGCCCGTGCCCTGGCCCACCCTC
GTGACCACCCTCGGCTACGGCCTGCAGTGCTTCGCCCGCTACCCCGACCACATGAAGCAGCACGACTTCTTCAAGTCCGC
CATGCCCGAAGGCTACGTCCAGGAGCGCACCATCTTCTTCAAGGACGACGGCAACTACAAGACCCGCGCCGAGGTGAAGT
TCGAGGGCGACACCCTGGTGAACCGCATCGAGCTGAAGGGCATCGACTTCAAGGAGGACGGCAACATCCTGGGGCACAA
GCTGGAGTACAACTACAACAGCCACAACGTCTATATCACCGCCGACAAGCAGAAGAACGGCATCAAGGCCAACTTCAAGAT
CCGCCACAACATCGAGGACGGCGGCGTGCAGCTCGCCGACCACTACCAGCAGAACACCCCCATCGGCGACGGCCCCGT
GCTGCTGCCCGACAACCACTACCTGAGCTACCAGTCCAAGCTGAGCAAAGACTAATCTAGAGGGCCCGTTTAAACCCGCT
GATCAGCCTCGACTGTGCCTTCTAGTTGCCAGCCATCTGTTGTTTGCCCCTCCCCCGTGCCTTCCTTGACCCTGGAAGGTG
CCACTCCCACTGTCCTTTCCTAATAAAATGAGGAAATTGCATCGCATTGTCTGAGTAGGTGTCATTCTATTCTGGGGGGTGG
GGTGGGGCAGGACAGCAAGGGGGAGGATTGGGAAGACAATAGCAGGCATGCTGGGGATGCGGTGGGCTCTATGGCTTCT
GAGGCGGAAAGAACCAGCTGGGGCTCTAGGGGGTATCCCCACGCGCCCTGTAGCGGCGCATTAAGCGCGGCGGGTGTG
GTGGTTACGCGCAGCGTGACCGCTACACTTGCCAGCGCCCTAGCGCCCGCTCCTTTCGCTTTCTTCCCTTCCTTTCTCGCC
ACGTTCGCCGGCTTTCCCCGTCAAGCTCTAAATCGGGGGCTCCCTTTAGGGTTCCGATTTAGTGCTTTACGGCACCTCGAC
CCCAAAAAACTTGATTAGGGTGATGGTTCACGTAGTGGGCCATCGCCCTGATAGACGGTTTTTCGCCCTTTGACGTTGGAG
TCCACGTTCTTTAATAGTGGACTCTTGTTCCAAACTGGAACAACACTCAACCCTATCTCGGTCTATTCTTTTGATTTATAAGG
GATTTTGCCGATTTCGGCCTATTGGTTAAAAAATGAGCTGATTTAACAAAAATTTAACGCGAATTAATTCTGTGGAATGTGTG
TCAGTTAGGGTGTGGAAAGTCCCCAGGCTCCCCAGCAGGCAGAAGTATGCAAAGCATGCATCTCAATTAGTCAGCAACCA
GGTGTGGAAAGTCCCCAGGCTCCCCAGCAGGCAGAAGTATGCAAAGCATGCATCTCAATTAGTCAGCAACCATAGTCCCG
CCCCTAACTCCGCCCATCCCGCCCCTAACTCCGCCCAGTTCCGCCCATTCTCCGCCCCATGGCTGACTAATTTTTTTTATTT
ATGCAGAGGCCGAGGCCGCCTCTGCCTCTGAGCTATTCCAGAAGTAGTGAGGAGGCTTTTTTGGAGGCCTAGGCTTTTGC
AAAAAGCTCCCGGGAGCTTGTATATCCATTTTCGGATCTGATCAGCACGTGATGAAAAAGCCTGAACTCACCGCGACGTCT
GTCGAGAAGTTTCTGATCGAAAAGTTCGACAGCGTCTCCGACCTGATGCAGCTCTCGGAGGGCGAAGAATCTCGTGCTTTC
AGCTTCGATGTAGGAGGGCGTGGATATGTCCTGCGGGTAAATAGCTGCGCCGATGGTTTCTACAAAGATCGTTATGTTTAT
CGGCACTTTGCATCGGCCGCGCTCCCGATTCCGGAAGTGCTTGACATTGGGGAATTCAGCGAGAGCCTGACCTATTGCAT
CTCCCGCCGTGCACAGGGTGTCACGTTGCAAGACCTGCCTGAAACCGAACTGCCCGCTGTTCTGCAGCCGGTCGCGGAG
GCCATGGATGCGATCGCTGCGGCCGATCTTAGCCAGACGAGCGGGTTCGGCCCATTCGGACCGCAAGGAATCGGTCAAT
ACACTACATGGCGTGATTTCATATGCGCGATTGCTGATCCCCATGTGTATCACTGGCAAACTGTGATGGACGACACCGTCA
GTGCGTCCGTCGCGCAGGCTCTCGATGAGCTGATGCTTTGGGCCGAGGACTGCCCCGAAGTCCGGCACCTCGTGCACGC
GGATTTCGGCTCCAACAATGTCCTGACGGACAATGGCCGCATAACAGCGGTCATTGACTGGAGCGAGGCGATGTTCGGGG
ATTCCCAATACGAGGTCGCCAACATCTTCTTCTGGAGGCCGTGGTTGGCTTGTATGGAGCAGCAGACGCGCTACTTCGAG
CGGAGGCATCCGGAGCTTGCAGGATCGCCGCGGCTCCGGGCGTATATGCTCCGCATTGGTCTTGACCAACTCTATCAGAG
CTTGGTTGACGGCAATTTCGATGATGCAGCTTGGGCGCAGGGTCGATGCGACGCAATCGTCCGATCCGGAGCCGGGACT
GTCGGGCGTACACAAATCGCCCGCAGAAGCGCGGCCGTCTGGACCGATGGCTGTGTAGAAGTACTCGCCGATAGTGGAA
ACCGACGCCCCAGCACTCGTCCGAGGGCAAAGGAATAGCACGTGCTACGAGATTTCGATTCCACCGCCGCCTTCTATGAA
AGGTTGGGCTTCGGAATCGTTTTCCGGGACGCCGGCTGGATGATCCTCCAGCGCGGGGATCTCATGCTGGAGTTCTTCGC
CCACCCCAACTTGTTTATTGCAGCTTATAATGGTTACAAATAAAGCAATAGCATCACAAATTTCACAAATAAAGCATTTTTTTC
ACTGCATTCTAGTTGTGGTTTGTCCAAACTCATCAATGTATCTTATCATGTCTGTATACCGTCGACCTCTAGCTAGAGCTTGG
CGTAATCATGGTCATAGCTGTTTCCTGTGTGAAATTGTTATCCGCTCACAATTCCACACAACATACGAGCCGGAAGCATAAA
GTGTAAAGCCTGGGGTGCCTAATGAGTGAGCTAACTCACATTAATTGCGTTGCGCTCACTGCCCGCTTTCCAGTCGGGAAA
CCTGTCGTGCCAGCTGCATTAATGAATCGGCCAACGCGCGGGGAGAGGCGGTTTGCGTATTGGGCGCTCTTCCGCTTCCT
CGCTCACTGACTCGCTGCGCTCGGTCGTTCGGCTGCGGCGAGCGGTATCAGCTCACTCAAAGGCGGTAATACGGTTATCC
ACAGAATCAGGGGATAACGCAGGAAAGAACATGTGAGCAAAAGGCCAGCAAAAGGCCAGGAACCGTAAAAAGGCCGCGTT

GCTGGCGTTTTTCCATAGGCTCCGCCCCCCTGACGAGCATCACAAAAATCGACGCTCAAGTCAGAGGTGGCGAAACCCGA
CAGGACTATAAAGATACCAGGCGTTTCCCCCTGGAAGCTCCCTCGTGCGCTCTCCTGTTCCGACCCTGCCGCTTACCGGAT
ACCTGTCCGCCTTTCTCCCTTCGGGAAGCGTGGCGCTTTCTCATAGCTCACGCTGTAGGTATCTCAGTTCGGTGTAGGTCG
TTCGCTCCAAGCTGGGCTGTGTGCACGAACCCCCCGTTCAGCCCGACCGCTGCGCCTTATCCGGTAACTATCGTCTTGAG
TCCAACCCGGTAAGACACGACTTATCGCCACTGGCAGCAGCCACTGGTAACAGGATTAGCAGAGCGAGGTATGTAGGCGG
TGCTACAGAGTTCTTGAAGTGGTGGCCTAACTACGGCTACACTAGAAGAACAGTATTTGGTATCTGCGCTCTGCTGAAGCC
AGTTACCTTCGGAAAAAGAGTTGGTAGCTCTTGATCCGGCAAACAAACCACCGCTGGTAGCGGTTTTTTTGTTTGCAAGCA
GCAGATTACGCGCAGAAAAAAAGGATCTCAAGAAGATCCTTTGATCTTTTCTACGGGGTCTGACGCTCAGTGGAACGAAAA
CTCACGTTAAGGGATTTTGGTCATGAGATTATCAAAAAGGATCTTCACCTAGATCCTTTTAAATTAAAAATGAAGTTTTAAATC
AATCTAAAGTATATATGAGTAAACTTGGTCTGACAGTTACCAATGCTTAATCAGTGAGGCACCTATCTCAGCGATCTGTCTAT
TTCGTTCATCCATAGTTGCCTGACTCCCCGTCGTGTAGATAACTACGATACGGGAGGGCTTACCATCTGGCCCCAGTGCTG
CAATGATACCGCGAGAACCACGCTCACCGGCTCCAGATTTATCAGCAATAAACCAGCCAGCCGGAAGGGCCGAGCGCAGA
AGTGGTCCTGCAACTTTATCCGCCTCCATCCAGTCTATTAATTGTTGCCGGGAAGCTAGAGTAAGTAGTTCGCCAGTTAATA
GTTTGCGCAACGTTGTTGCCATTGCTACAGGCATCGTGGTGTCACGCTCGTCGTTTGGTATGGCTTCATTCAGCTCCGGTT
CCCAACGATCAAGGCGAGTTACATGATCCCCCATGTTGTGCAAAAAAGCGGTTAGCTCCTTCGGTCCTCCGATCGTTGTCA
GAAGTAAGTTGGCCGCAGTGTTATCACTCATGGTTATGGCAGCACTGCATAATTCTCTTACTGTCATGCCATCCGTAAGATG
CTTTTCTGTGACTGGTGAGTACTCAACCAAGTCATTCTGAGAATAGTGTATGCGGCGACCGAGTTGCTCTTGCCCGGCGTC
AATACGGGATAATACCGCGCCACATAGCAGAACTTTAAAAGTGCTCATCATTGGAAAACGTTCTTCGGGGCGAAAACTCTCA
AGGATCTTACCGCTGTTGAGATCCAGTTCGATGTAACCCACTCGTGCACCCAACTGATCTTCAGCATCTTTTACTTTCACCA
GCGTTTCTGGGTGAGCAAAAACAGGAAGGCAAAATGCCGCAAAAAAGGGAATAAGGGCGACACGGAAATGTTGAATACTC
ATACTCTTCCTTTTTCAATATTATTGAAGCATTTATCAGGGTTATTGTCTCATGAGCGGATACATATTTGAATGTATTTAGAAA
AATAAACAAATAGGGGTTCCGCGCACATTTCCCCGAAAAGTGCCACCTGACGTC

CRISPR/dCas9-VenN

Annotated features:

SV40 NLS

Linker

dCas9

VenN

Full plasmid sequence:

TGGAAGGGCTAATTCACTCCCAAAGAAGACAAGATATCCTTGATCTGTGGATCTACCACACACAAGGCTACTTCCCTGATTA
GCAGAACTACACACCAGGGCCAGGGGTCAGATATCCACTGACCTTTGGATGGTGCTACAAGCTAGTACCAGTTGAGCCAG
ATAAGGTAGAAGAGGCCAATAAAGGAGAGAACACCAGCTTGTTACACCCTGTGAGCCTGCATGGGATGGATGACCCGGAG
AGAGAAGTGTTAGAGTGGAGGTTTGACAGCCGCCTAGCATTTCATCACGTGGCCCGAGAGCTGCATCCGGAGTACTTCAA
GAACTGCTGATATCGAGCTTGCTACAAGGGACTTTCCGCTGGGGACTTTCCAGGGAGGCGTGGCCTGGGCGGGACTGGG
GAGTGGCGAGCCCTCAGATCCTGCATATAAGCAGCTGCTTTTTGCCTGTACTGGGTCTCTCTGGTTAGACCAGATCTGAGC
CTGGGAGCTCTCTGGCTAACTAGGGAACCCACTGCTTAAGCCTCAATAAAGCTTGCCTTGAGTGCTTCAAGTAGTGTGTGC
CCGTCTGTTGTGTGACTCTGGTAACTAGAGATCCCTCAGACCCTTTTAGTCAGTGTGGAAAATCTCTAGCAGTGGCGCCCG
AACAGGGACTTGAAAGCGAAAGGGAAACCAGAGGAGCTCTCTCGACGCAGGACTCGGCTTGCTGAAGCGCGCACGGCAA
GAGGCGAGGGGCGGCGACTGGTGAGTACGCCAAAAATTTTGACTAGCGGAGGCTAGAAGGAGAGAGATGGGTGCGAGAG
CGTCAGTATTAAGCGGGGGAGAATTAGATCGCGATGGGAAAAAATTCGGTTAAGGCCAGGGGGAAAGAAAAAATATAAATT
AAAACATATAGTATGGGCAAGCAGGGAGCTAGAACGATTCGCAGTTAATCCTGGCCTGTTAGAAACATCAGAAGGCTGTAG
ACAAATACTGGGACAGCTACAACCATCCCTTCAGACAGGATCAGAAGAACTTAGATCATTATATAATACAGTAGCAACCCTC
TATTGTGTGCATCAAAGGATAGAGATAAAAGACACCAAGGAAGCTTTAGACAAGATAGAGGAAGAGCAAAACAAAAGTAAG
ACCACCGCACAGCAAGCGGCCGGCCGCTGATCTTCAGACCTGGAGGAGGAGATATGAGGGACAATTGGAGAAGTGAATTA
TATAAATATAAAGTAGTAAAAATTGAACCATTAGGAGTAGCACCCACCAAGGCAAAGAGAAGAGTGGTGCAGAGAGAAAAAA
GAGCAGTGGGAATAGGAGCTTTGTTCCTTGGGTTCTTGGGAGCAGCAGGAAGCACTATGGGCGCAGCGTCAATGACGCTG
ACGGTACAGGCCAGACAATTATTGTCTGGTATAGTGCAGCAGCAGAACAATTTGCTGAGGGCTATTGAGGCGCAACAGCAT
CTGTTGCAACTCACAGTCTGGGGCATCAAGCAGCTCCAGGCAAGAATCCTGGCTGTGGAAAGATACCTAAAGGATCAACAG
CTCCTGGGGATTTGGGGTTGCTCTGGAAAACTCATTTGCACCACTGCTGTGCCTTGGAATGCTAGTTGGAGTAATAAATCTC
TGGAACAGATTTGGAATCACACGACCTGGATGGAGTGGGACAGAGAAATTAACAATTACACAAGCTTAATACACTCCTTAAT
TGAAGAATCGCAAAACCAGCAAGAAAAGAATGAACAAGAATTATTGGAATTAGATAAATGGGCAAGTTTGTGGAATTGGTTT
AACATAACAAATTGGCTGTGGTATATAAAATTATTCATAATGATAGTAGGAGGCTTGGTAGGTTTAAGAATAGTTTTTGCTGT
ACTTTCTATAGTGAATAGAGTTAGGCAGGGATATTCACCATTATCGTTTCAGACCCACCTCCCAACCCCGAGGGGACCCGA
CAGGCCCGAAGGAATAGAAGAAGAAGGTGGAGAGAGAGACAGAGACAGATCCATTCGATTAGTGAACGGATCTCGACGGT
ATCGCCGAATTGACAAATGGCAGTATTCATCCACAATTTTAAAAGAAAAGGGGGGATTGGGGGGTACAGTGCAGGGGAAAG
AATAGTAGACATAATAGCAACAGACATACAAACTAAAGAATTACAAAAACAAATTACAAAAATTCAAAATTTTCGGGTTTATTA
CAGGGACAGCAGAGATCCAGTTTGGACTAGCGATGCTAGCTGATGCGGGCCCTAGTTATTAATAGTAATCAATTACGGGGT
CATTAGTTCATAGCCCATATATGGAGTTCCGCGTTACATAACTTACGGTAAATGGCCCGCCTGGCTGACCGCCCAACGACC
CCCGCCCATTGACGTCAATAATGACGTATGTTCCCATAGTAACGCCAATAGGGACTTTCCATTGACGTCAATGGGTGGAGT
ATTTACGGTAAACTGCCCACTTGGCAGTACATCAAGTGTATCATATGCCAAGTACGCCCCCTATTGACGTCAATGACGGTAA
ATGGCCCGCCTGGCATTATGCCCAGTACATGACCTTATGGGACTTTCCTACTTGGCAGTACATCTACGTATTAGTCATCGCT

ATTACCATGGTGATGCGGTTTTGGCAGTACATCAATGGGCGTGGATAGCGGTTTGACTCACGGGGATTTCCAAGTCTCCAC
CCCATTGACGTCAATGGGAGTTTGTTTTGGCACCAAAATCAACGGGACTTTCCAAAATGTCGTAACAACTCCGCCCCATTGA
CGCAAATGGGCGGTAGGCGTGTACGGTGGGAGGTCTATATAAGCAGAGCTCTCCCTATCAGTGATAGAGATCTCCCTATCA
GTGATAGAGATCGTCGACGAGCTCGTTTAGTGAACCGTCAGATCGCCTGGAGACGCCATCCACGCTGTTTTGACCTCCATA
GAAGACACCGGGACCGATCCAGCCTACCGGTTAAGGTGAACGCGTGCAGGCTGGCGCCACCATGGCCCCTAAAAAGAAA
CGCAAGGTCGAATTCGGAAGCGGAAGCGACAAGAAGTACTCCATTGGGCTCGCTATCGGTACCAACAGCGTCGGCTGGG
CCGTCATTACGGACGAGTACAAGGTGCCGAGCAAAAAATTCAAAGTTCTGGGCAATACCGATCGCCACAGCATAAAGAAGA
ACCTCATTGGAGCCCTCCTGTTCGACTCCGGGGAGACGGCCGAAGCCACGCGGCTCAAAAGAACAGCACGGCGCAGATA
TACCCGCAGAAAGAATCGGATCTGCTACCTGCAGGAGATCTTTAGTAATGAGATGGCTAAGGTGGATGACTCTTTCTTCCAT
AGGCTGGAGGAGTCCTTTTTGGTGGAGGAGGATAAAAAGCACGAGCGCCACCCAATCTTTGGCAATATCGTGGACGAGGT
GGCGTACCATGAAAAGTACCCAACCATATATCATCTGAGGAAGAAGCTGGTAGACAGTACTGATAAGGCTGACTTGCGGTT
GATCTATCTCGCGCTGGCGCACATGATCAAATTTCGGGGACACTTCCTCATCGAGGGGGACCTGAACCCAGACAACAGCG
ATGTCGACAAACTCTTTATCCAACTGGTTCAGACTTACAATCAGCTTTTCGAGGAGAACCCGATCAACGCATCCGGCGTTGA
CGCCAAAGCAATCCTGAGCGCTAGGCTGTCCAAATCCCGGCGGCTCGAAAACCTCATCGCACAGCTCCCTGGGGAGAAGA
AGAACGGCCTGTTTGGTAATCTTATCGCCCTGTCACTCGGGCTGACCCCCAACTTTAAATCTAACTTCGACCTGGCCGAAG
ATGCCAAGCTGCAACTGAGCAAAGACACCTACGATGATGATCTCGACAATCTGCTGGCCCAGATCGGCGACCAGTACGCA
GACCTTTTTTTGGCGGCAAAGAACCTGTCAGACGCCATTCTGCTGAGTGATATTCTGCGAGTGAACACGGAGATCACCAAA
GCTCCGCTGAGCGCTAGTATGATCAAGCGCTATGATGAGCACCACCAAGACTTGACTTTGCTGAAGGCCCTTGTCAGACAG
CAACTGCCTGAGAAGTACAAGGAAATTTTCTTCGATCAGTCTAAAAATGGCTACGCCGGATACATTGACGGCGGAGCAAGC
CAGGAGGAATTTTACAAATTTATTAAGCCCATCTTGGAAAAAATGGACGGCACCGAGGAGCTGCTGGTAAAGCTGAACAGA
GAAGATCTGTTGCGCAAACAGCGCACTTTCGACAATGGAAGCATCCCCCACCAGATTCACCTGGGCGAACTGCACGCTATC
CTCAGGCGGCAAGAGGATTTCTACCCCTTTTTGAAAGATAACAGGGAAAAGATTGAGAAAATCCTCACATTTCGGATACCCT
ACTATGTAGGCCCCCTCGCTCGGGGAAATTCCAGATTCGCGTGGATGACTCGCAAATCAGAAGAGACCATCACTCCCTGG
AACTTCGAGGAAGTCGTGGATAAGGGGGCCTCTGCCCAGTCCTTCATCGAAAGGATGACTAACTTTGATAAAAATCTGCCT
AACGAAAAGGTGCTTCCTAAACACTCTCTGCTGTACGAGTACTTCACAGTTTATAACGAGCTCACCAAGGTCAAATACGTCA
CAGAAGGGATGAGAAAGCCAGCATTCCTGTCTGGAGAGCAGAAGAAAGCTATCGTGGACCTCCTCTTCAAGACGAACCGG
AAAGTTACCGTGAAACAGCTCAAAGAAGACTATTTCAAAAAGATTGAATGTTTCGACTCTGTTGAAATCAGCGGAGTGGAGG
ATCGCTTCAACGCATCCCTGGGAACGTATCACGATCTCCTGAAAATCATTAAAGACAAGGACTTCCTGGACAATGAGGAGA
ACGAGGACATTCTTGAGGACATTGTCCTCACCCTTACGTTGTTTGAAGATAGGGAGATGATTGAAGAACGCTTGAAAACTTA
CGCTCATCTCTTCGACGACAAAGTCATGAAACAGCTCAAGAGACGCCGATATACAGGATGGGGGCGGCTGTCAAGAAAAC
TGATCAATGGCATCCGAGACAAGCAGAGTGGAAAGACAATCCTGGATTTTCTTAAGTCCGATGGATTTGCCAACCGGAACT
TCATGCAGTTGATCCATGATGACTCTCTCACCTTTAAGGAGGACATCCAGAAAGCACAAGTTTCTGGCCAGGGGGACAGTC
TTCACGAGCACATCGCTAATCTTGCAGGTAGCCCAGCTATCAAAAAGGGAATACTGCAGACCGTTAAGGTCGTGGATGAAC
TCGTCAAAGTAATGGGAAGGCATAAGCCCGAGAATATCGTTATCGAGATGGCCCGAGAGAACCAAACTACCCAGAAGGGA
CAGAAGAACAGTAGGGAAAGGATGAAGAGGATTGAAGAGGGTATAAAAGAACTGGGGTCCCAAATCCTTAAGGAACACCC
AGTTGAAAACACCCAGCTTCAGAATGAGAAGCTCTACCTGTACTACCTGCAGAACGGCAGGGACATGTACGTGGATCAGGA
ACTGGACATCAACCGGTTGTCCGACTACGACGTGGATGCTATCGTGCCCCAAAGCTTTCTCAAAGATGATTCTATTGATAAT
AAAGTGTTGACAAGATCCGATAAAAATAGAGGGAAGAGTGATAACGTCCCCTCAGAAGAAGTTGTCAAGAAAATGAAAAATT
ATTGGCGGCAGCTGCTGAACGCCAAACTGATCACACAACGGAAGTTCGATAATCTGACTAAGGCTGAACGAGGTGGCCTG
TCTGAGTTGGATAAAGCCGGCTTCATCAAAAGGCAGCTTGTTGAGACACGCCAGATCACCAAGCACGTGGCCCAAATTCTC
GATTCACGCATGAACACCAAGTACGATGAAAATGACAAACTGATTCGAGAGGTGAAAGTTATTACTCTGAAGTCTAAGCTGG
TCTCAGATTTCAGAAAGGACTTTCAGTTTTATAAGGTGAGAGAGATCAACAATTACCACCATGCGCATGATGCCTACCTGAA
TGCAGTGGTAGGCACTGCACTTATCAAAAAATATCCCAAGCTGGAATCTGAATTTGTTTACGGAGACTATAAAGTGTACGAT
GTTAGGAAAATGATCGCAAAGTCTGAGCAGGAAATAGGCAAGGCCACCGCTAAGTACTTCTTTTACAGCAATATTATGAATT
TTTTCAAGACCGAGATTACACTGGCCAATGGAGAGATTCGGAAGCGACCACTTATCGAAACAAACGGAGAAACAGGAGAAA
TCGTGTGGGACAAGGGTAGGGATTTCGCGACAGTCCGCAAGGTCCTGTCCATGCCGCAGGTGAACATCGTTAAAAAGACC
GAAGTACAGACCGGAGGCTTCTCCAAGGAAAGTATCCTCCCGAAAAGGAACAGCGACAAGCTGATCGCACGCAAAAAAGA
TTGGGACCCCAAGAAATACGGCGGATTCGATTCTCCTACAGTCGCTTACAGTGTACTGGTTGTGGCCAAAGTGGAGAAAGG
GAAGTCTAAAAAACTCAAAAGCGTCAAGGAACTGCTGGGCATCACAATCATGGAGCGATCCAGCTTCGAGAAAAACCCCAT
CGACTTTCTCGAAGCGAAAGGATATAAAGAGGTCAAAAAAGACCTCATCATTAAGCTGCCCAAGTACTCTCTCTTTGAGCTT
GAAAACGGCCGGAAACGAATGCTCGCTAGTGCGGGCGAGCTGCAGAAAGGTAACGAGCTGGCACTGCCCTCTAAATACGT
TAATTTCTTGTATCTGGCCAGCCACTATGAAAAGCTCAAAGGGTCTCCCGAAGATAATGAGCAGAAGCAGCTGTTCGTGGA
ACAACACAAACACTACCTTGATGAGATCATCGAGCAAATAAGCGAGTTCTCCAAAAGAGTGATCCTCGCCGACGCTAACCT
CGATAAGGTGCTTTCTGCTTACAATAAGCACAGGGATAAGCCCATCAGGGAGCAGGCAGAAAACATTATCCACTTGTTTACT
CTGACCAACTTGGGCGCGCCTGCAGCCTTCAAGTACTTCGACACCACCATAGACAGAAAGCGGTACACCTCTACAAAGGA
GGTCCTGGACGCCACACTGATTCATCAGTCAATTACGGGGCTCTATGAAACAAGAATCGACCTCTCTCAGCTCGGTGGAGA
CGGCTCTACTAGTGGCTCTCCCAAGAAGAAGAGGAAGGTAGGATCCGGAAGTATGGTGAGCAAGGGCGAGGAGCTGTTCA
CCGGGGTGGTGCCCATCCTGGTCGAGCTGGACGGCGACGTAAACGGCCACAAGTTCAGCGTGTCCGGCGAGGGCGAGG
GCGATGCCACCTACGGCAAGCTGACCCTGAAGCTCATCTGCACCACCGGCAAGCTGCCCGTGCCCTGGCCCACCCTCGT
GACCACCCTCGGCTACGGCCTGCAGTGCTTCGCCCGCTACCCCGACCACATGAAGCAGCACGACTTCTTCAAGTCCGCCA
TGCCCGAAGGCTACGTCCAGGAGCGCACCATCTTCTTCAAGGACGACGGCAACTACAAGACCCGCGCCGAGGTGAAGTTC
GAGGGCGACACCCTGGTGAACCGCATCGAGCTGAAGGGCATCGACTTCAAGGAGGACGGCAACATCCTGGGGCACAAGC
TGGAGTACAACTACAACAGCCACAACGTCTATATCACCGCCGACAAGCAGAAGAACGGCATCAAGGCCAACTTCAAGATCC
GCCACAACATCGAGGACGGCGGCGTGCAGCTCGCCGACCACTACCAGCAGAACACCCCCATCGGCGACGGCCCCGTGCT
GCTGCCCGACAACCACTACCTGAGCTACCAGTCCAAGCTGAGCAAAGACTAATCTAGATAATCAACCTCTGGATTACAAAAT
TTGTGAAAGATTGACTGGTATTCTTAACTATGTTGCTCCTTTTACGCTATGTGGATACGCTGCTTTAATGCCTTTGTATCATG
CTATTGCTTCCCGTATGGCTTTCATTTTCTCCTCCTTGTATAAATCCTGGTTGCTGTCTCTTTATGAGGAGTTGTGGCCCGTT
GTCAGGCAACGTGGCGTGGTGTGCACTGTGTTTGCTGACGCAACCCCCACTGGTTGGGGCATTGCCACCACCTGTCAGCT
CCTTTCCGGGACTTTCGCTTTCCCCCTCCCTATTGCCACGGCGGAACTCATCGCCGCCTGCCTTGCCCGCTGCTGGACAG
GGGCTCGGCTGTTGGGCACTGACAATTCCGTGGTGTTGTCGGGGAAATCATCGTCCTTTCCTTGGCTGCTCGCCTGTGTT
GCCACCTGGATTCTGCGCGGGACGTCCTTCTGCTACGTCCCTTCGGCCCTCAATCCAGCGGACCTTCCTTCCCGCGGCCT
GCTGCCGGCTCTGCGGCCTCTTCCGCGTCTTCGCCTTCGCCCTCAGACGAGTCGGATCTCCCTTTGGGCCGCCTCCCCGC

CTGAGATCCTTTAAGACCAATGACTTACAAGGCAGCTGTAGATCTTAGCCACTTTTTAAAAGAAAAGGGGGGACTGGAAGG
GCTAATTCACTCCCAACGAAGACAAGATCTGCTTTTTGCTTGTACTGGGTCTCTCTGGTTAGACCAGATCTGAGCCTGGGA
GCTCTCTGGCTAACTAGGGAACCCACTGCTTAAGCCTCAATAAAGCTTGCCTTGAGTGCTTCAAGTAGTGTGTGCCCGTCT
GTTGTGTGACTCTGGTAACTAGAGATCCCTCAGACCCTTTTAGTCAGTGTGGAAAATCTCTAGCAGTAGTAGTTCATGTCAT
CTTATTATTCAGTATTTATAACTTGCAAAGAAATGAATATCAGAGAGTGAGAGGCCCGGGTTAATTAAGGAAAGGGCTAGAT
CATTCTTGAAGACGAAAGGGCCTCGTGATACGCCTATTTTTATAGGTTAATGTCATGATAATAATGGTTTCTTAGACGTCAGG
TGGCACTTTTCGGGGAAATGTGCGCGGAACCCCTATTTGTTTATTTTTCTAAATACATTCAAATATGTATCCGCTCATGAGAC
AATAACCCTGATAAATGCTTCAATAATATTGAAAAAGGAAGAGTATGAGTATTCAACATTTCCGTGTCGCCCTTATTCCCTTT
TTTGCGGCATTTTGCCTTCCTGTTTTTGCTCACCCAGAAACGCTGGTGAAAGTAAAAGATGCTGAAGATCAGTTGGGTGCAC
GAGTGGGTTACATCGAACTGGATCTCAACAGCGGTAAGATCCTTGAGAGTTTTCGCCCCGAAGAACGTTTTCCAATGATGA
GCACTTTTAAAGTTCTGCTATGTGGCGCGGTATTATCCCGTGTTGACGCCGGGCAAGAGCAACTCGGTCGCCGCATACACT
ATTCTCAGAATGACTTGGTTGAGTACTCACCAGTCACAGAAAAGCATCTTACGGATGGCATGACAGTAAGAGAATTATGCAG
TGCTGCCATAACCATGAGTGATAACACTGCGGCCAACTTACTTCTGACAACGATCGGAGGACCGAAGGAGCTAACCGCTTT
TTTGCACAACATGGGGGATCATGTAACTCGCCTTGATCGTTGGGAACCGGAGCTGAATGAAGCCATACCAAACGACGAGC
GTGACACCACGATGCCTGTAGCAATGGCAACAACGTTGCGCAAACTATTAACTGGCGAACTACTTACTCTAGCTTCCCGGC
AACAATTAATAGACTGGATGGAGGCGGATAAAGTTGCAGGACCACTTCTGCGCTCGGCCCTTCCGGCTGGCTGGTTTATTG
CTGATAAATCTGGAGCCGGTGAGCGTGGGTCTCGCGGTATCATTGCAGCACTGGGGCCAGATGGTAAGCCCTCCCGTATC
GTAGTTATCTACACGACGGGGAGTCAGGCAACTATGGATGAACGAAATAGACAGATCGCTGAGATAGGTGCCTCACTGATT
AAGCATTGGTAACTGTCAGACCAAGTTTACTCATATATACTTTAGATTGATTTAAAACTTCATTTTTAATTTAAAAGGATCTAG
GTGAAGATCCTTTTTGATAATCTCATGACCAAAATCCCTTAACGTGAGTTTTCGTTCCACTGAGCGTCAGACCCCGTAGAAA
AGATCAAAGGATCTTCTTGAGATCCTTTTTTTCTGCGCGTAATCTGCTGCTTGCAAACAAAAAAACCACCGCTACCAGCGGT
GGTTTGTTTGCCGGATCAAGAGCTACCAACTCTTTTTCCGAAGGTAACTGGCTTCAGCAGAGCGCAGATACCAAATACTGTT
CTTCTAGTGTAGCCGTAGTTAGGCCACCACTTCAAGAACTCTGTAGCACCGCCTACATACCTCGCTCTGCTAATCCTGTTAC
CAGTGGCTGCTGCCAGTGGCGATAAGTCGTGTCTTACCGGGTTGGACTCAAGACGATAGTTACCGGATAAGGCGCAGCGG
TCGGGCTGAACGGGGGGTTCGTGCACACAGCCCAGCTTGGAGCGAACGACCTACACCGAACTGAGATACCTACAGCGTG
AGCTATGAGAAAGCGCCACGCTTCCCGAAGGGAGAAAGGCGGACAGGTATCCGGTAAGCGGCAGGGTCGGAACAGGAGA
GCGCACGAGGGAGCTTCCAGGGGGAAACGCCTGGTATCTTTATAGTCCTGTCGGGTTTCGCCACCTCTGACTTGAGCGTC
GATTTTTGTGATGCTCGTCAGGGGGGCGGAGCCTATGGAAAAACGCCAGCAACGCGGCCTTTTTACGGTTCCTGGCCTTTT
GCTGGCCTTTTGCTCACATGTTCTTTCCTGCGTTATCCCCTGATTCTGTGGATAACCGTATTACCGCCTTTGAGTGAGCTGA
TACCGCTCGCCGCAGCCGAACGACCGAGCGCAGCGAGTCAGTGAGCGAGGAAGCGGAAGAGCGCCCAATACGCAAACC
GCCTCTCCCCGCGCGTTGGCCGATTCATTAATGCAGCAAGCTCATGGCTGACTAATTTTTTTTATTTATGCAGAGGCCGAG
GCCGCCTCGGCCTCTGAGCTATTCCAGAAGTAGTGAGGAGGCTTTTTTGGAGGCCTAGGCTTTTGCAAAAAGCTCCCCGT
GGCACGACAGGTTTCCCGACTGGAAAGCGGGCAGTGAGCGCAACGCAATTAATGTGAGTTAGCTCACTCATTAGGCACCC
CAGGCTTTACACTTTATGCTTCCGGCTCGTATGTTGTGTGGAATTGTGAGCGGATAACAATTTCACACAGGAAACAGCTATG
ACATGATTACGAATTTCACAAATAAAGCATTTTTTTCACTGCATTCTAGTTGTGGTTTGTCCAAACTCATCAATGTATCTTATC
ATGTCTGGATCAACTGGATAACTCAAGCTAACCAAAATCATCCCAAACTTCCCACCCCATACCCTATTACCACTGCCAATTA
CCTGTGGTTTCATTTACTCTAAACCTGTGATTCCTCTGAATTATTTTCATTTTAAAGAAATTGTATTTGTTAAATATGTACTACA
AACTTAGTAGT

Supplementary Figure 27: Uncropped versions of all gel images. The approximate regions of the cropped area as shwon in the prepared figures is indicated with a blue box.

Supplementary Figure 5c

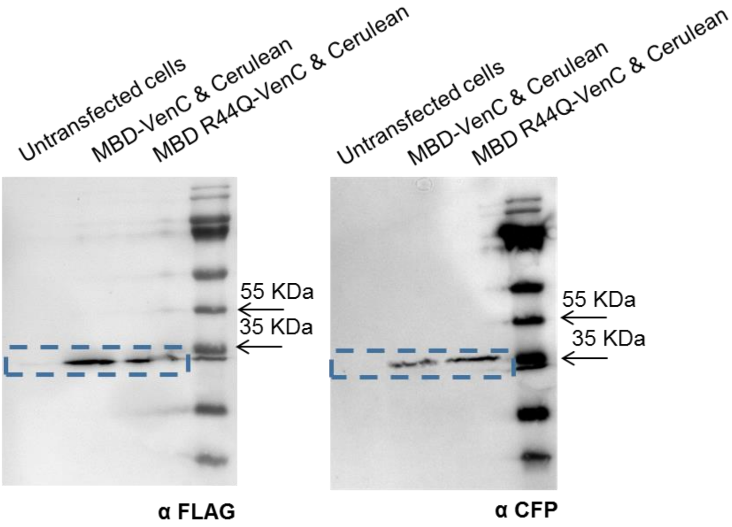

Supplementary Figure 16a

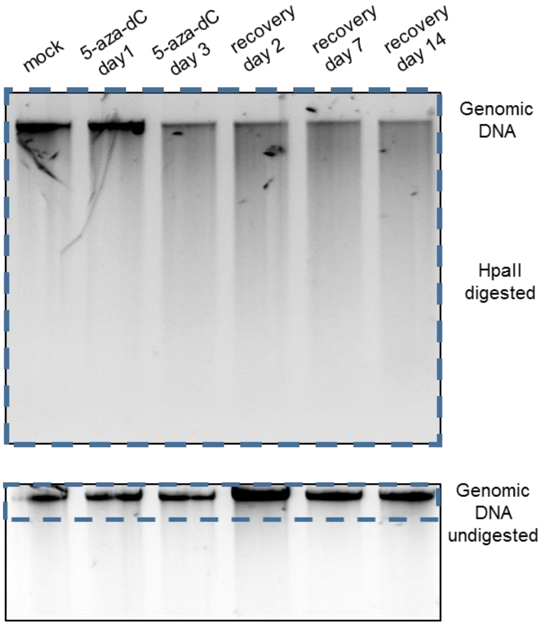

The position of the gel loading dye front and periodic checking under the UV lap were used to decide when to stop the gel. No size marker was used here.

Supplementary Figure 17a

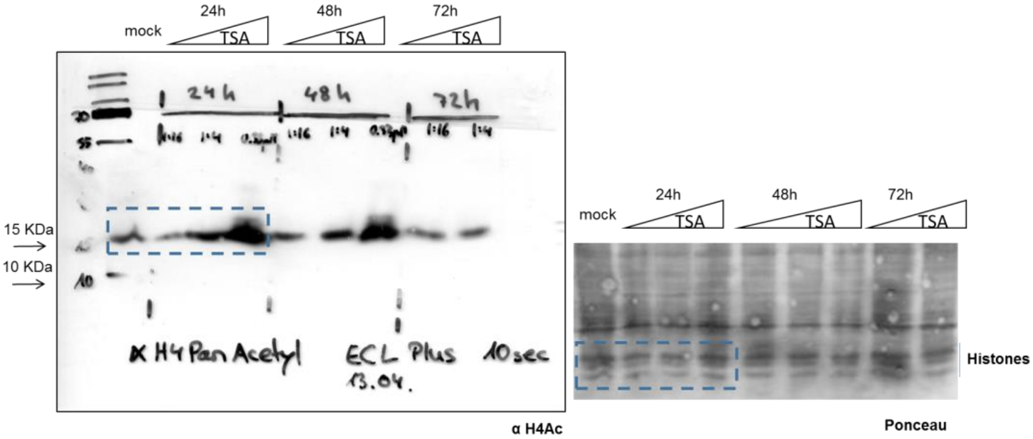

Supplementary Figure 17b

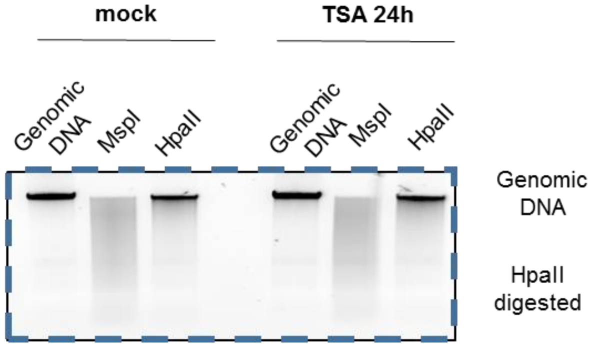

Supplementary Figure 24c

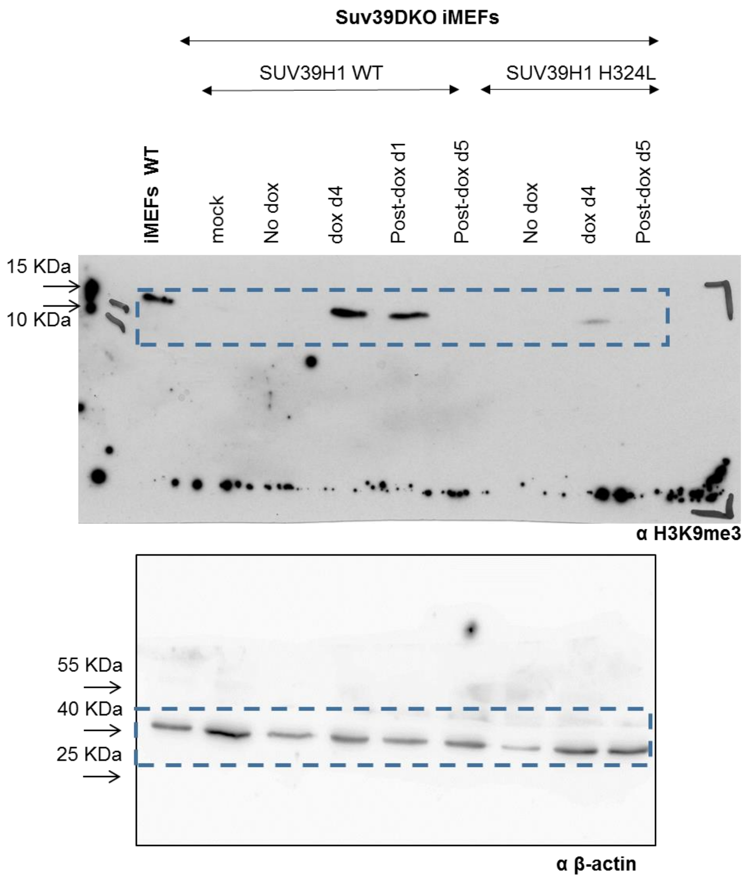

Supplementary Figure 25b

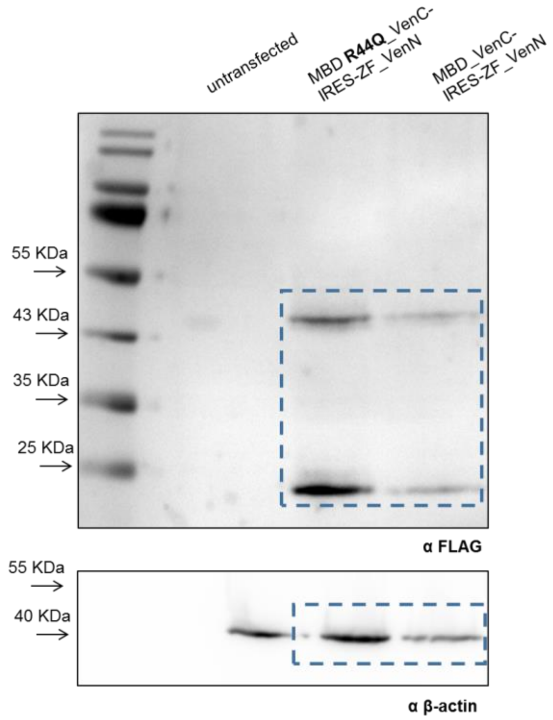

Supplemental Tables

Supplementary Table 1: BiFC assays setup for the BiAD sensor 1

Cell line	Transfection reagent	Total plasmid amount [ng]	ZF anchor amount [ng]	MBD detector amount [ng]	Related to Figure #
NIH 3T3	Fugene HD	2000	50	50	Fig. 2b
iMEF WT	GenaxxonFect	2500	50	50	Fig. 2a, d; Supplementary Figs.3, 4, 6 and 9
iMEF Suv39DKO	GenaxxonFect	2500	50	50	Supplementary Fig. 9
iMEF *P53*$^{-/-}$	Lipofectamine 3000	2500	50	50	Fig. 2c; Supplementary Fig. 3b
iMEF *P53*$^{-/-}$/*Dnmt1*$^{-/-}$	Lipofectamine 3000	2500	50	50	Fig. 2c, d; Supplementary Figs. 3b and 8

Supplementary Table 2: BiFC assays setup for the BiAD sensor 2

Cell line	Transfection reagent	Total plasmid amount [ng]	TALE anchor amount [ng]	MBD detector amount [ng]	Related to Figure #
HEK293	Fugene HD	2000	50	500	Figs. 3, 4d, e and Supplementary Figs. 3b, 12, 13b, 14, 17c and d;
HCT116 WT	Lipofectamine 3000	2500	100	1000	Fig. 4a, c
HCT116 *DNMT1*$^{-/-}$	Lipofectamine 3000	2500	100	1000	Fig. 4b, c

Supplementary Table 3: BiFC assays setup for the BiAD sensor 3

Cell line	Transfection reagent	Total plasmid amount [ng]	dCas9 anchor amount [ng]	guide RNA amount [ng]	MBD detector amount [ng]	Related to Figure #
HEK293	Fugene HD	2000	250	750	250	Fig.5 and Supplementary Fig. 19

Supplementary Table 4: BiFC assays setup for the BiAD sensor 4

Cell line	Transfection reagent	Total plasmid amount [ng]	ZF anchor amount [ng]	HP1CD detector amount [ng]	Related to Figure #
iMEF WT	GenaxxonFect	2500	250	750	Fig. 6a, c and Supplementary Figs. 22, 23c, d
iMEF Suv39DKO	GenaxxonFect	2500	250	750	Fig. 6b, c; and Supplementary Fig. 22a
iMEF Suv39DKO + Tet-SUV39H1 WT	GenaxxonFect	2500	250	750	Fig. 6b, c
iMEF Suv39DKO + Tet-SUV39H1 H324L	GenaxxonFect	2500	250	750	Fig. 6b, c

Supplementary Table 5: Settings used for fluorophore imaging

Fluorophore	Lase line for excitation [nm]	Emission collection window [nm]
DAPI/mCerulean	405	418-573
LSSmKate2	488	595-731
mVenus/ BiFC	514	525-602
Alexa 594/ mRuby2/ mCherry	561	595-731

Supplementary Table 6: Number of counted cells for graphs shown in Figures 2-7 & Supplementary Figs. 9, 17 and 23

BiAD sensor	Cell line	Detector domain variant	Drug treatment/ Exogenous expression of epigenetic enzyme	Number of counted cells per replicate	Related to Figure #
1	iMEF WT	MBD WT	not treated	20-30	Fig. 2d
		MBD R44Q	not treated	20-30	
	iMEF *P53*$^{-/-}$/*Dnmt1*$^{-/-}$	MBD WT	not treated	20-30	
	iMEF WT	MBD WT	not treated	15-20	Supplementary Fig. 9b
	iMEF Suv39DKO	MBD WT	not treated	15-20	
**1,** stable integration	MBD_VenC-IRES-ZF_VenN	MBD WT	5-aza-dC/ mock	25-45 per time point	Fig. 7c
2	HEK293	MBD WT	not treated	40-60	Fig. 3d
	HEK293	MBD R44Q	not treated	40-60	
	HEK293	MBD WT	5-aza-dC/ mock	20-60 per time point	Fig. 4c
	HEK293	MBD WT	mock	15-20	Supplementary Fig. 17d
	HEK293	MBD WT	TSA	15-20	
	HCT116 WT	MBD WT	not treated	20-30	Fig. 4e
	HCT116 *DNMT1*$^{-/-}$	MBD WT	not treated	25-35	
	HCT116 *DNMT1*$^{-/-}$	MBD WT	DNMT1 WT	20-30	
	HCT116 *DNMT1*$^{-/-}$	MBD WT	DNMT1 C1226A	20-40	
3	HEK293	MBD WT	not treated	15-20	Fig. 5d
	HEK293	MBD R44Q	not treated	15-20	
	HEK293	MBD WT	5-aza-dC/ mock	15-20 per time point	Fig. 5f
4	iMEF WT	HP1CD WT	not treated	20-30	Fig. 6c
	iMEF Suv39DKO	HP1CD WT	not treated	20-30	
	iMEF Suv39DKO + Tet-SUV39H1 WT	HP1CD WT	Tet-SUV39H1 WT	15-20	
	iMEF Suv39DKO + Tet-SUV39H1 H324L	HP1CD WT	Tet-SUV39H1 H324L	15-20	
	iMEF WT	HP1CD WT	not treated	20-30	Supplementary Fig. 23d
		HP1CD W42A	not treated	20-30	

Supplementary Table 7: p values for graphs shown in Figures 2-7 & Supplementary Figs. 9, 17 and 23

BiAD sensor	BiAD variants compared	Cell line background compared	p value	Related to Figure #
**1**	MBD WT vs MBD R44Q	iMEFs	**0.0028**	Fig. 2d
	MBD WT	iMEF WT vs iMEF *P53*$^{-/-}$/*Dnmt1*$^{-/-}$	**0.0033**	
	MBD WT	iMEF WT vs iMEF Suv39DKO	0.1601	Supplementary Fig. 9b
**1,** stable integration	MBD WT	Mock vs 5-aza-dC (day 3)	**0.0006**	Fig. 7c
	MBD WT	Mock vs 5-aza-dC (recovery day 1)	**0.0023**	
**2**	MBD WT vs MBD R44Q	HEK293	**0.0043**	Fig. 3d
	MBD WT	HCT116 WT vs HCT116 *DNMT1*$^{-/-}$	**0.0022**	Fig. 4c
	MBD WT	HCT116 *DNMT1*$^{-/-}$ vs HCT116 *DNMT1*$^{-/-}$ + DNMT1 WT	**0.0047**	
	MBD WT	HCT116 *DNMT1*$^{-/-}$ vs HCT116 *DNMT1*$^{-/-}$ + DNMT1 C1226A	0.2285	
	MBD WT	Mock vs 5-aza-dC (day 3)	**0.0017**	Fig. 4e
	MBD WT	Mock vs 5-aza-dC (recovery day 14)	**0.0110**	
	MBD WT	Mock vs TSA	0.0766	Supplementary Fig. 17d
**3**	MBD WT vs MBD R44Q	HEK293	**0.0017**	Fig. 5d
	MBD WT	Mock vs 5-aza-dC (day 3)	**0.0001**	Fig. 5f
**4**	HP1CD WT	iMEF WT vs iMEF Suv39DKO	**0.0012**	Fig. 6c
	HP1CD WT	iMEF Suv39DKO vs iMEF Suv39DKO +Tet-SUV39H1 WT	**0.0136**	
	HP1CD WT	iMEF Suv39DKO vs iMEF Suv39DKO + Tet-SUV39H1 H324L	0.2712	
	HP1CD WT vs HP1CD W42A	iMEF	**0.0009**	Supplementary Fig. 23d
* Comparisons with p > 0.05 are considered not significant. p-values < 0.05 are printed in bold.				

Supplementary Table 8: Primers used for amplicon-targeted bisulfite sequencing

Cell line	Fw primer (5' – 3´)	Rv primer (5' – 3´)
HEK293	CAGATCGTTATTGGATATTTGGAGTATTTTGAGGTTTATGG	CAGTACAAACCATCCTAACTAACAAAATAAAACCC
HCT 116	ACATGTGTTATTGGATATTTGGAGTATTTTGAGGTTTATGG	ACTAGTAAACCATCCTAACTAACAAAATAAAACCC
HCT116 *DNMT1*$^{-/-}$	CTTGTAGTTATTGGATATTTGGAGTATTTTGAGGTTTATGG	CTGTTAAAACCATCCTAACTAACAAAATAAAACCC
Underlined nucleotides denote the barcodes used for sample identification.		

Supplementary references

1 Arand, J. *et al.* In vivo control of CpG and non-CpG DNA methylation by DNA methyltransferases. *PLoS genetics* **8**, e1002750, doi:10.1371/journal.pgen.1002750 (2012).

2 Lu, L. Y., Kuang, H., Korakavi, G. & Yu, X. C. Topoisomerase II Regulates the Maintenance of DNA Methylation. *Journal of Biological Chemistry* **290**, 851-860, doi:10.1074/jbc.M114.611509 (2015).

3 Ricci, M. A., Manzo, C., Garcia-Parajo, M. F., Lakadamyali, M. & Cosma, M. P. Chromatin fibers are formed by heterogeneous groups of nucleosomes in vivo. *Cell* **160**, 1145-1158, doi:10.1016/j.cell.2015.01.054 (2015).
